# Holding the Belief That Gender Roles Can Change Reduces Women’s Work–Family Conflict

**DOI:** 10.1177/01461672231178349

**Published:** 2023-06-18

**Authors:** Charlotte H. Townsend, Laura J. Kray, Alexandra G. Russell

**Affiliations:** 1 University of California, Berkeley, USA; 2 Stanford University, CA, USA

**Keywords:** gender roles, mindsets, work–family conflict, gender differences, COVID-19

## Abstract

Across four studies (*N* = 1544), we examined the relationship between individuals’ gender role mindsets, or beliefs about the malleability versus fixedness of traditional gender roles, and work–family conflict. We found that undergraduate women (but not men) business students holding a fixed, compared to growth, gender role mindset anticipated more work–family conflict. Next, we manipulated gender role mindset and demonstrated a causal link between women’s growth mindsets (relative to fixed mindsets and control conditions) and reduced work–family conflict. We showed mechanistically that growth gender role mindsets unburden women from prescriptive gender roles, reducing work–family conflict. Finally, during COVID-19, we demonstrated a similar pattern among working women in high-achieving dual-career couples. We found an indirect effect of women’s gender role mindset on job and relationship satisfaction, mediated through work–family conflict. Our preregistered studies suggest that holding the belief that gender roles can change mitigates women’s work–family conflict.

Consistently over the last several years, recent corporate surveys revealed that working women are more likely than their men counterparts to think about downshifting their careers or leaving the workforce, and the women who do stay are experiencing higher rates of burnout (Thomas et al., 2020, 2022a, 2022b). The COVID-19 pandemic has further exacerbated a long-standing conflict between women’s paid work and personal lives, with women spending increasingly more time on childcare and household tasks (Giurge et al., 2021). For example, research has shown that school closures during COVID-19 directly reduced women’s work productivity (Ain Tommar et al., 2022). Given the prevalence of these conflicts between work and home in women’s lives, which result in reduced satisfaction and burnout, it is imperative to identify levers to mitigate their psychological toll.

Interrole conflict occurs in response to opposing pressures to participate in different social roles (Kahn et al., 1964). Work–family conflict, a form of interrole conflict, is a subjective feeling that personal and professional goals are in conflict and contributes to the loss of women in the paid labor economy (Fouad et al., 2011; Greenhaus & Beutell, 1985). Work–family conflict is intensified by competing demands between traditional gender roles and “ideal worker” expectations (van der Lippe & Lippényi, 2020). Ideal worker expectations include employees being devoted to their jobs, working long hours as needed, and rearranging their personal lives around paid work (Kelly et al., 2010). Traditional gender roles prescribe that women provide greater than equal contributions at home. Despite increasing their share of the labor workforce, women, many of whom work full-time jobs, are still responsible for most household tasks, including family responsibilities (Coltrane, 2000). Given that traditional gender roles prescribe that men should be the primary earners and have a less central role in caregiving and household work than women, men tend to have less work–family conflict (Duxbury & Higgins, 1991). By contrast, the competing demands between work and the gendered expectations of domestic labor for women result in relatively more work–family conflict. This tension between work and family tends to increase among women over their lifetime (Hall, 1975) and is often resolved by women reducing their commitment to paid work or quitting altogether (Kim, 2020; Rosen & Jerdee, 1974).

Another important consequence of work–family conflict is reduced satisfaction. Prior literature has established a strong relationship between work–family conflict and both work and family satisfaction (Ernst Kossek & Ozeki, 1998; Ford et al., 2007; Lee et al., 2014; Martins et al., 2002), especially for women in men-dominated fields (Settles, 2004). Unfortunately, women’s reduced satisfaction in these domains also contributes to their choosing less-demanding careers or leaving the workforce entirely (Becker, 1985; Thomas et al., 2020). Between 2019 and 2021, during the COVID-19 pandemic, the global workforce lost 13 million women; by contrast, men’s employment has recovered to pre-COVID levels (International Labour Organization, 2021).

## The Effect of Gender Roles on Work–Family Conflict

The term *gender roles* refers to divisions of household labor, job segregation, and gender differences in status and authority. Gender roles influence how people are expected to act, speak, dress, and conduct themselves based on their gender (Basow, 1992). Traditional gender roles, which assign women to take care of the home and men to provide financial resources, are powerful because they not only describe historical labor distributions but also dictate which roles men and women *should* assume (Eagly & Karau, 2002; Prentice & Carranza, 2002; Wood & Eagly, 2012).

Beliefs about gender roles can also have significant behavioral consequences (Schmader et al., 2004; Tiedemann, 2000; Williams et al., 2010 etc.). Girls who endorse gender role stereotypes perform worse and are more likely to withdraw from men-dominated fields (Schmader et al., 2004; Steffens et al., 2010). Further, more traditional gender role beliefs predict preferences for stereotypical occupations and gender-stereotypical wage gaps, as well as greater perceived disparities in what men and women are thought to earn, thus bolstering the gender pay gap (Tinsley et al., 2015; Williams et al., 2010). Given the restrictive nature of gender roles, we propose that beliefs that gender roles can change may help reduce women’s reported work–family conflict by relaxing prescriptions that require women to fulfill domestic responsibilities.

## The Role of Gender Role Mindsets in Work–Family Conflict

Dweck’s (2006) influential theory on mindsets states that people hold implicit theories about the malleability of attributes in themselves and others (Dweck et al., 1995; Dweck & Leggett, 1988). Those with fixed mindsets believe that individuals’ basic qualities are stable and unchanging, while those with growth mindsets believe that individuals’ basic abilities or characteristics are malleable and amenable to change. Across contexts and situations, fixed mindsets are associated with a greater focus on outcomes, achievement, and image and increased reliance on stereotypes. Growth mindsets, by contrast, are associated with a strong orientation toward learning and growth and a greater tendency to update perceptions of self and others using new and relevant information instead of stereotypes (Chiu et al., 1997; Levy et al., 1998; Mueller & Dweck, 1998; Plaks et al., 2005). Mindsets have been investigated across various contexts: They have been applied to individual characteristics such as intelligence, personality, and negotiating skills, and they have also been applied to social groups in organizational contexts (Blackwell et al., 2007; Canning et al., 2020; Kray & Haselhuhn, 2007; Rattan & Dweck, 2018; Rattan & Ozgumus, 2019). Despite their subjective nature, espousing a growth mindset has proven to improve behavioral performance measures, such as negotiation outcomes, scholastic achievement, and confronting bias.

Recent work has broadened the scope of mindset research, exploring implications for gendered divisions of labor and opportunities in society (Kray et al., 2017). *Gender role mindset* reflects implicit beliefs about the mutability versus fixedness of gender roles in society. Individuals with a fixed gender role mindset hold the belief that the sorting of men and women into distinct social roles is unchangeable, with women’s (vs. men’s) responsibility for the domestic (vs. financial) sphere deemed immutable. By contrast, individuals with a growth gender role mindset hold the belief that these social roles are changeable, such that women need not be primarily responsible for providing domestic support and men need not be primarily responsible for providing financial support. Given the varied consequences of prescriptive gender roles on how men’s and women’s lives unfold, we are interested in examining how mindsets about gender roles influence work–family conflict.

In the present research, we examined whether gender role mindsets are an especially strong predictor of work–family conflict in women. We expected that, if women hold the belief that social roles cannot change, the conflict between work and family may seem inescapable because gender role prescriptions dictate that women should prioritize family over work. Conversely, we expected that espousing the belief that gender roles are changeable may reduce women’s work–family conflict by relaxing their need to fulfill prescriptive gender roles. Unburdened from traditional gender roles, women may feel liberated to scale back on caregiving duties to reduce conflict between work and home demands, instead of feeling that they have to scale back at work to be consistent with traditional gender roles.

Given that traditional gender roles dictate that men should be the primary earners, and fixed mindsets promote the status quo (Kray et al., 2017), which advantages men in the workplace, we did not expect fixed gender role mindsets to be similarly tied to work–family conflict for men. When they hold more egalitarian gender ideologies, men contribute more to housework and childcare and are also more likely to take advantage of parental leave (Duvander, 2014; Grunow & Baur, 2014; Kroska, 2004). While one might hypothesize that holding a growth gender role mindset could increase men’s work–family conflict, we did not expect this to be the case. If gender role mindsets impact adherence to prescriptive gender roles, then a growth mindset might liberate men to scale back on work if they desire, but it would not serve as a mandate to do so. Indeed, a growth gender mindset would be expected to free both men and women from the prescriptive element of gender roles; as such, it should provide both men and women more freedom to choose what is best for them and their partner as they navigate the competing demands of work and family.

## The Present Research

Across four studies, we examined how gender role mindset relates to work–family conflict in women. While past research has shown that women face more tradeoffs than men when choosing to enter the workforce (Budig & England, 2001), for the first time, we examined whether this conflict is mitigated for women holding a growth gender role mindset. Study 1 examined anticipated work–family conflict among young adults. In Study 2, we conducted an experiment with a novel manipulation involving self-generated examples of how gender roles remain fixed versus are changing. This allowed us to test the causal role of growth gender role mindset in reducing work–family conflict for women and examine the directionality of work–family conflict (i.e., work demands interfering with home versus home demands interfering with work). In Study 3, we added a control condition to clarify the direction of the effects of gender role mindset on work–family conflict. We also tested a potential mechanism and showed that growth mindsets lead to less prescriptive gender role adherence in women.

Study 4 examined the link between gender role mindset and work–family conflict in dual-career couples, along with downstream consequences in terms of relationship and job satisfaction. All couples participated in our study during the COVID-19 pandemic, between April and November 2020. Prior research has demonstrated the relevance of mindsets, specifically in challenging situations, such as the COVID-19 pandemic. The pandemic was also a relationship stressor, making this an important context to measure satisfaction (Pietromonaco & Overall, 2021). Dual-career couples were forced to navigate work demands and household responsibilities, making this an ideal setting in which to explore the effect of gender role mindsets on work–family conflict.

All preregistrations, data, and code are available for review (https://osf.io/p6mey/).

## Study 1

The first study examined whether there is a relationship between gender role mindsets and anticipated work–family conflict. We expected this conflict between professional and personal goals to be intensified for fixed gender role mindset women. Conversely, since traditional gender roles support men being career oriented, we predicted that men with a fixed gender role mindset will exhibit less or no perceived conflict between work and personal life. To explore this relationship in undergraduate students, we measured their anticipated work–family conflict.

### Method

#### Participants

We recruited *n* = 162 undergraduates at a large research university who completed a general prescreening that would make them eligible for future studies and would partially fulfill a course requirement.^1^ Thus, sample size was determined by course enrollment. All participants in our study completed the survey during September 2015. See Table 1 for participant demographic information across studies.

**Table 1.** Demographics for Participants in Studies 1 to 4.

Demographic categories	Study 1 (*N* = 162)	Study 2 (*N* = 483)	Study 3 (*N* = 703)	Study 4 (*N* = 196)
Gender				
Woman	85 (52.5%)	483 (100.0%)	483 (100.0%)	98 (50.0%)
Man	77 (47.5%)	0 (0.0%)	0 (0.0%)	98 (50.0%)
Employment status				
Employed full time	n/a	211 (43.7%)	320 (45.5%)	172 (87.8%)
Employed part time	n/a	64 (13.3%)	98 (13.9%)	24 (12.3%)
Unemployed	n/a	42 (8.7%)	43 (6.1%)	n/a
Self-employed	n/a	58 (12.0%)	68 (9.7%)	n/a
Homemaker	n/a	51 (10.6%)	61 (8.7%)	n/a
Student	162 (100%)	10 (2.1%)	63 (9.0%)	n/a
Retired	n/a	47 (9.7%)	50 (7.1%)	n/a
Ethnicity				
White/Caucasian	38 (23.5%)	385 (79.7%)	540 (76.8%)	86 (43.9%)
Asian/Asian American	84 (51.9%)	28 (5.8%)	44 (6.3%)	101 (51.5%)
Black/African American	5 (3.1%)	31 (6.4%)	62 (8.8%)	3 (1.5%)
Hispanic/Latino	15 (9.3%)	23 (4.8%)	31 (4.4%)	11 (5.6%)
East Asian/East Asian American	1 (0.6%)	0 (0.0%)	4 (0.6%)	n/a
South Asian	8 (4.9%)	n/a	n/a	n/a
Middle Eastern	3 (1.9%)	n/a	n/a	6 (3.1%)
Native/American Indian	n/a	n/a	n/a	1 (0.5%)
Multiracial	n/a	12 (2.5%)	19 (2.7%)	n/a
Other	8 (4.9%)	4 (0.8%)	3 (0.4%)	2 (1.0%)
Age				
*M* (*SD*)	22.0 (3.7)	43.1 (14.4)	40.8 (14.7)	n/a
Median [min, max]	21.3 [19.8, 49.9]	41.0 [19.0, 93.0]	38.0 [18.0, 83.0]	n/a

*Note.* Participants did not report employment status in Study 1, but all participants were undergraduate students. In Study 4, participants chose between the following employment options: “Full-time,” “75%–99% time,” “50%–74% time,” “25%–49% time,” “1%–24% time,” and “Other” (no one selected “Other”). Participants could also select more than one category for ethnicity could be selected, and age was reported in categorical age groups.

#### Measures and Procedure

Participants were given a link to an online survey that was part of a broader study. The following subset of measures were our measures of interest (see Supplemental Material for items):

##### Gender Role Mindset

Participants completed Kray et al.’s (2017) 10-item gender role mindset measure (α = .88). Items are “I think that men and women will always have different social roles,” “Even though it’s not always popular to say so, men and women will always inhabit different roles in society,” “I think that men are suited for different roles than women,” “It’s only a matter of time before men and women will inhabit the same social roles” (reverse-scored), “Even though I might not want to admit it, men and women naturally hold different positions in society,” “I don’t think there’s any innate reason for men and women to have different roles in society” (reverse-scored), “No matter how much society progresses, differences in the societal roles of women and men will persist,” “As society progresses, men and women will eventually occupy similar roles in society” (reverse-scored), “It’s only a matter of time before men and women will be fulfilling the same societal roles” (reverse-scored), and “Both men and women are well-suited for most societal roles” (reverse-scored). Responses were measured on a scale ranging from 1 (*strongly disagree*) to 6 (*strongly agree*), with higher scores indicating greater endorsement of fixed mindsets.

##### Anticipated Work–Family Conflict

Participants completed a 7-item measure of the amount of work–family conflict they believed they would experience in the future. We constructed this measure to tap general conflict between personal and professional domains, but not the directionality of the conflict, as this was not central to our hypotheses. Our items include “I anticipate no conflict between achieving both my personal and professional goals in life” (reverse-scored), “At some point, I expect to have to choose between a fulfilling home life and a successful professional life,” “Having a family will mean sacrificing my career,” “Pursuing a rewarding career will mean sacrificing in my personal life,” “I feel conflicted between pursuing my career aspirations as well as my personal aspirations,” “I don’t expect to be able to have it all when it comes to personal and professional accomplishments,” and “I don’t see any reason why I can’t have both a prestigious career and a fulfilling personal life” (reverse-scored). The response scale ranged from 1 (*disagree entirely*) to 7 (*agree entirely*). Higher scores indicated greater anticipated work–family conflict (α = .78).

##### Control Variables

###### Biological Essentialism

Participants indicated their agreement with a 7-item measure (α = .83) of gender-specific biological essentialism (BE) (Brescoll et al., 2013, adapted from Keller, 2005). Sample item includes “Men commit the majority of violent crimes in this country because they have a greater predisposition toward violence than women.” The response scale ranged from 1 (*strongly disagree*) to 6 (*strongly agree*). Higher scores indicated greater endorsement of essentialist explanations of gender differences (Haslam et al., 2000), which might cause work–family conflict for women working outside of the home as doing so violates biological imperatives for child-rearing.

###### Preference for Traditional Gender Roles

Participants completed Larsen and Long’s (1988) 20-item Attitudes Toward Sex Roles Scale (α = .90). Participants rated how much they agreed with eight egalitarian belief statements (e.g., “Having a job is just as important for a wife as it is for her husband”) and 12 traditional belief statements (e.g., “Women should be more concerned with clothing and appearance than men”). Response scales ranged from 1 (*strongly disagree*) to 6 (*strongly agree*). The eight egalitarian statements were reverse-scored, and all items were averaged with higher values indicating a stronger personal preference for traditional gender roles (PTGR), which might cause work–family conflict for women working outside of the home if they prefer to restrict their work to within the home.

### Results

Table 2 reports descriptive statistics and correlations for all variables.^2^ We included as predictors gender, gender role mindset, endorsement of BE, and PTGR, along with the interaction of gender and each individual difference measure. Table 3 summarizes the results of the linear regression predicting anticipated work–family conflict.

**Table 2.** Descriptive Statistics and Correlations for Study 1 Variables.

	Men (*N* = 77)		Women (*N* = 85)		Correlations				
Variable	*M*	*SD*	*M*	*SD*	(1)	(2)	(3)	(4)	(5)
(1) Gender	—	—	—	—	—				
(2) Work–family conflict	3.8	1.1	4.1	1.0	.14†	—			
(3) Gender role mindset	3.4	0.7	3.3	0.7	−.10	.20*	—		
(4) Biological essentialism	3.5	1.0	3.4	0.8	−.07	.17*	.30***	—	
(5) Traditional gender role preference	2.4	0.9	2.1	0.9	−.15†	.08	.34***	.49***	—

*Note.* Gender role mindset: Higher values indicate more fixed mindset.

† *p* < .10. *p < .05. **p < .01. ***p < .001.

**Table 3.** Study 1 Regression Analysis of Anticipated Work–Family Conflict.

Predictors	Model 1					Model 2					Model 3					Model 4				
	β	*SE*	CI	*t*	*p* value	β	*SE*	CI	*t*	*p* value	β	*SE*	CI	*t*	*p* value	β	*SE*	CI	*t*	*p* value
Gender	.33	0.15	[0.02, 0.63]	2.13	.**035**	.32	0.15	[0.02, 0.62]	2.10	.**037**	.31	0.15	[0.01, 0.62]	2.05	.**042**	.32	0.15	[0.01, 0.62]	2.05	.**042**
GRM	.21	0.08	[0.06, 0.36]	2.75	.**007**	.02	0.12	[−0.20, 0.25]	0.20	.843	−.03	0.12	[−0.26, 0.21]	−0.21	.832	−.04	0.12	[−0.27, 0.20]	−0.30	.768
Gender × GRM						.34	0.15	[0.04, 0.64]	2.21	.**028**	.37	0.15	[0.06, 0.67]	2.37	.**019**	.41	0.17	[0.08, 0.73]	2.45	.**015**
PTGR											−.06	0.09	[−0.24, 0.12]	−0.64	.526	.02	0.13	[−0.23, 0.28]	0.19	.849
BE											.17	0.09	[−0.00, 0.35]	1.93	.056	.15	0.12	[−0.09, 0.39]	1.20	.234
Gender × PTGR																−.17	0.18	[−0.53, 0.19]	−0.92	.360
Gender × BE																.04	0.18	[−0.32, 0.39]	0.21	.834
Observations	162					162					161					161				
*R*^2^/*R*^2^ adjusted	.065/.053					.093/.076					.114/.085					.119/.079				

*Note.* Gender coded. 0 = man, 1 = woman. Anticipated work–family conflict, GRM, PTGR, and BE measures were all standardized. CI = confidence interval; GRM = gender role mindset; PTGR = preference for traditional gender roles; BE = biological essentialism.

Bolded *p*-values are statistically significant (*p* < .05).

**Figure 1.**
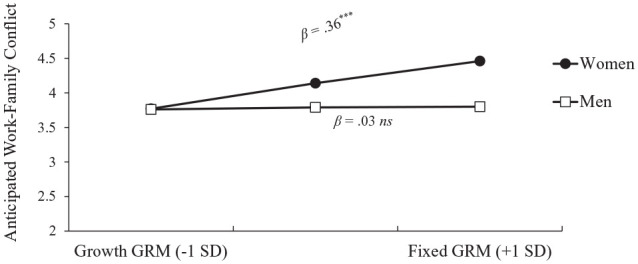
Anticipated Work–Family Conflict by Gender and GRM in Undergraduates (Study 1). *Note.* GRM = gender role mindset. †*p* < .10. *p < .05. **p < .01. ***p < .001.

Three effects emerged as significant. First, relative to men (*M* = 3.8, *SD* = 1.1), women anticipated more work–family conflict (*M* = 4.1, *SD* = 1.0), β = .33, *t*(159) = 2.13, *p* = .035, *f* = .15, 95% confidence interval (CI) = [0.0, 0.30]. Second, a fixed gender role mindset predicted more anticipated work–family conflict than a growth gender role mindset, β = .21, *t*(159) = 2.75, *p* = .007, *f* = .22, 95% CI = [0.06, 0.38]. Finally, the Gender × Gender Role Mindset interaction emerged, depicted in Figure 1, β = .34, *t*(158) = 2.21, *p* = .028, *f* = .18, 95% CI = [0.02, 0.33]. For women, a fixed gender role mindset predicted more anticipated work–family conflict, β = .36, *t*(158) = 3.55, *p* < .001; for men, gender role mindset did not predict anticipated work–family conflict, β = .03, *t*(158) = 0.20, *p* = .84. Comparing participants with a fixed (+1 *SD*) gender role mindset, women had significantly more anticipated work–family conflict than men, β = .66, *t*(158) = 3.09, *p* = .002. However, women with a growth (−1 *SD*) gender role mindset did not significantly differ in anticipated work–family conflict from men with a growth gender role mindset, β = −.02, *t*(158) = −0.08, *p* = .937. This effect of gender role mindset on anticipated work–family conflict for women remained significant when including control measures in the model: PTGR, endorsement of BE, and interaction terms with gender (see Table 3 Model 4).

### Discussion

We found initial evidence of a relationship between gender role mindset and anticipated work–family conflict. Women anticipated greater work–family conflict than men, and women with a more fixed gender role mindset anticipated greater work–family conflict than women with a growth gender role mindset. Finally, our interaction of interest was significant: A more fixed gender role mindset is correlated with anticipated work–family conflict for women, but not men.

## Study 2

In Study 1, we demonstrated a correlational relationship between gender role mindset and anticipated work–family conflict. In Study 2, we demonstrated the causal relationship between gender role mindsets and our work–family conflict measure. We created a manipulation of gender role mindsets based on previous work that has used a self-reflection exercise, rather than a scientific article, to shift mindsets (Heslin et al., 2005; Wilson, 1990).^3^ We also extended this effect to a well-established work–family conflict scale that distinguishes between conflict experienced when work impinges on domestic responsibilities versus family impinges on work responsibilities (Netemeyer et al., 1996), controlling for additional implicit attitudes. Our goal was to demonstrate the convergent validity of our holistic measure of work–family conflict with preexisting measures from the organizational behavior literature. In addition, we included new control measures, gender determinism, and implicit personality mindset, to further disentangle our distinct measure from other constructs.

### Method

We preregistered that women in the fixed condition would report more work–family conflict than women in the growth condition.^4^

#### Participants

We recruited *n* = 500 online women participants in the United States from Prolific who self-identified as heterosexual and ended up with 500 participants when the study completed.

#### Measures and Procedure

Participants were given a link to the online survey in which they were assigned to reflect and write about either how gender roles have changed or how they have persisted (see Supplemental Material for exact wording). We asked participants to complete the Netemeyer et al. (1996) work–family conflict and family–work conflict subscales. We did not have a directional prediction about which aspect of interrole conflict would be affected by gender role mindsets. Next, participants also completed gender determinism and implicit personality control measures.

##### Manipulation Check

Participants indicated, “To what degree have gender roles changed versus remained the same over time?” from 1 (*gender roles have remained the same*) to 7 (*gender roles have changed a lot*). This item was then reverse-coded so that higher values indicate more fixed beliefs.

##### Work–Family Conflict

Participants completed the same 7-item scale as Study 1 but worded in the present tense (α = .90).

##### Work–Family Conflict Subscale

Participants indicated their agreement with a 5-item measure (α = .96) of work–family conflict (Netemeyer et al., 1996). Sample item includes “The demands of my work interfere with my home and family life.” The response scale ranged from 1 (*strongly disagree*) to 7 (*strongly agree*). Higher scores indicated greater work–family conflict.

##### Family–Work Conflict Subscale

Participants indicated their agreement with a 5-item measure (α = .96) of family–work conflict (Netemeyer et al., 1996). Sample item includes “The demands of my family or spouse/partner interfere with work-related activities.” The response scale ranged from 1 (*strongly disagree*) to 7 (*strongly agree*). Higher scores indicated greater family–work conflict.

##### Control Variables

###### Gender Determinism

Participants indicated their agreement with a 4-item measure (α = .93) of gender determinism (Tinsley et al., 2015). Sample item includes “A person’s gender is something basic about them that determines how they will act.” The response scale ranged from 1 (*strongly do not believe*) to 5 (*strongly believe*). Higher scores indicated greater endorsement of gender determinism, which might cause work–family conflict for women working outside of the home if one believes gender categories dictate individual characteristics.

###### Implicit Personality Mindset

Participants indicated their agreement with an 8-item measure (α = .95) of implicit personality mindset (Levy et al., 1998). Sample item includes “People can’t really change their most basic attributes.” The response scale ranged from 1 (*strongly disagree*) to 6 (*strongly agree*). Higher scores indicated more fixed implicit personality mindsets, which is an alternative explanation to our measure of mindsets specific to gender roles.

### Results

#### Manipulation Check

We found a significant difference between conditions, such that participants in the fixed condition (*M* = 3.93, *SD* = 1.41) more strongly believed that gender roles have remained unchanged than growth condition participants, *M* = 2.36, *SD* = 1.20, *t*(474) = 13.22, *p* < .001, *d* = 1.20, 95% CI = [1.01, 1.39].

#### Control Measures

We did not find a significant difference between conditions for gender determinism, *t*(481) = 1.78, *p* = .08, or implicit personality mindset, *t*(477) = 1.47, *p* = .14.

#### Work–Family Conflict

**Figure 2.**
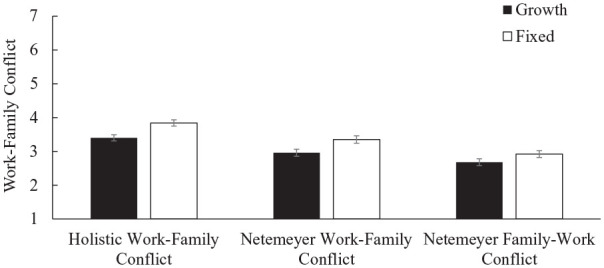
Effect of Condition on Women’s Work–Family Conflict Measures (Study 2). *Note.* Error bars represent standard errors.

Table 4 reports correlations for all study variables. We predicted work–family conflict by condition using a linear regression. We found a main effect of condition, such that women in the growth condition (*M* = 3.40, *SD* = 1.40) reported significantly less work–family conflict than in the fixed condition (*M* = 3.84, *SD* = 1.44), β = −.31, *t*(481) = −3.45, *p* < .001, *d* = .31, 95% CI = [0.13, 0.49]. See Figure 2. The effect remained significant when gender determinism and implicit personality were included and without participant exclusions, β = −.27, *t*(496) = −3.15, *p* = .002.^5^

**Table 4.** Correlations for Study 2 Variables.

	Correlations—growth condition					Correlations—fixed condition				
Variable	(1)	(2)	(3)	(4)	(5)	(1)	(2)	(3)	(4)	(5)
(1) Work–family conflict	—					—				
(2) Netemeyer work–family subscale	.61***	—				.49***	—			
(3) Netemeyer family–work subscale	.59***	.55***	—			.53***	.64***	—		
(4) Gender determinism	.23***	.10	.07	—		.15*	.09	.12†	—	
(5) Implicit personality mindset	.10	.01	−.01	.49***	—	.22***	.05	.07	.34***	—

† *p* < .10. *p < .05. **p < .01. ***p < .001.

We predicted Netemeyer’s work–family conflict and family–work conflict subscales by condition using linear regressions. We found a main effect of condition, such that women in the growth condition (*M* = 2.96, *SD* = 1.61; *M* = 2.68, *SD* = 1.55) reported significantly less work–family conflict and marginally less family–work conflict than in the fixed condition (*M* = 3.35, *SD* = 1.73; *M* = 2.92, *SD* = 1.57), β = −.23, *t*(481) = −2.54, *p* = .011, *d* = .23, 95% CI = [0.05, 0.41]; β = −.15, *t*(481) = −1.68, *p* = .093, *d* = .15, 95% CI = [−0.03, 0.33]. The effect of mindset condition on the work–family subscale remained significant when gender determinism and implicit personality were included and without participant exclusions, β = −.22, *t*(496) = −2.48, *p* = .013.

### Discussion

In this study, we demonstrated the causal relationship between gender role mindsets and our holistic measure of work–family conflict. Thus, this study provides further evidence that self-reflection is an effective method for manipulating gender role mindsets. As such, the present study contributes a novel gender role mindset manipulation to the literature. We also replicated the causal relationship between gender role mindsets and both Netemeyer’s well-established work–family and family–work conflict subscales. Our measure of work–family conflict varies from the Netemeyer subscales, in that it does not contend with the direction of the conflict. By including these subscales, we can explore this directionality and find evidence that conflict stems both from work interfering with family and family interfering with work. Finally, we showed that gender role mindset affects one’s level of work–family conflict, controlling for gender determinism and implicit personality mindset, suggesting the observed effects are not due to implicit beliefs about how deterministic gender is, nor the malleability of personalities more broadly.

## Study 3

In Study 2, we demonstrated the causal relationship between gender role mindsets and our holistic work–family conflict measure and extended this effect to a validated scale that distinguishes two potential directions of interrole conflict, controlling for additional implicit theories. In Study 3, we had two goals. First, we sought to establish the directionality of gender role mindsets’ effects on work–family conflict by adding a control condition to our experimental design. Second, we sought to explore a mechanism through which growth mindsets reduce women’s work–family conflict. Specifically, we described a scenario in which participants imagined experiencing a high degree of work–family conflict and examined the impact of the mindset manipulation on participants’ behavioral intentions to take actions to reduce it. Prior mindset literature has demonstrated that those with more fixed mindsets are more prone to stereotyping (Levy et al., 1998). Given that traditional gender role stereotypes imply that women should be responsible for duties in the home, we wanted to test whether gender role mindsets affect the felt prescriptiveness of gender roles (Prentice & Carranza, 2002). When faced with work–family conflict, women in the fixed mindset condition may feel intensified gender role prescriptions, evident in them believing they should scale back at work or become part time. In the growth mindset condition, women should feel more liberated from gender role prescriptions and thus more willing to reduce caregiving responsibilities or hire a nanny in response to work–family conflict situations.

### Method

Our experimental design included one gender role mindset factor with three levels (growth, fixed, control). We again preregistered that women in the fixed condition would report more work–family conflict than women in the growth condition. We did not have a directional hypothesis about the control condition.

#### Participants

We recruited *n* = 750 online women participants in the United States from Prolific who self-identified as heterosexual and ended up with 746 participants when the study completed.

#### Measures and Procedure

As in Study 2, participants were given a link to the online survey in which they were assigned to reflect and write about either how gender roles have changed or how they have persisted. In the control condition, participants were asked to write about gender roles, without specifying a focus on how they have changed or stayed the same. Varying from Studies 2, we next told participants the following:

Imagine that you are working at Tech. Inc, a company dedicated to making a better world. Your job is both engaging and demanding, requiring that you put in well over 40 hours per week. Your partner works full-time and you have two young children at home. The role pressures you are experiencing from the work and family domains are sources of stress. Something has to give.

We then asked participants how likely they would be to take various actions to reduce conflict. Next, participants completed our work–family conflict measure.

##### Manipulation Check

We used the same manipulation check as Study 2.

##### Behavioral Intentions

Participants indicated how likely they would be to take four different actions.^6^ Two items were designed to measure actions reflecting intensified prescriptions for women and two items were designed to measure actions reflecting relaxed prescriptions for women. We analyzed each item separately due to low reliability (α = .11). Items are “Cut back on my work responsibilities,” “Consider going part-time at work,” “Consider hiring a nanny,” and “Cut back on my caretaking responsibilities.” The response scale ranged from 1 (*extremely unlikely*) to 7 (*extremely likely*). Higher scores indicated a greater likelihood of taking said action.

##### Work–Family Conflict

Participants completed the same 7-item scale as Study 2 (α = .89).

### Results

#### Manipulation Check

We found a significant difference between conditions, *F*(2, 700) = 80.74, *p* < .001, η^2^ = .19, 95% CI = [0.14, 0.24], such that participants in the fixed condition (*M* = 3.86, *SD* = 1.27) more strongly believed that gender roles have remained unchanged than the control, *M* = 2.91, *SD* = 1.41; *t*(700) = 8.14, *p* < .001, and growth condition, *M* = 2.40, *SD* = 1.08; *t*(700) = 12.50, *p* < .001, participants.^7^ The difference between the growth and control conditions was also significant, *t*(700) = 4.35, *p* < .001.

#### Work–Family Conflict

**Figure 3.**
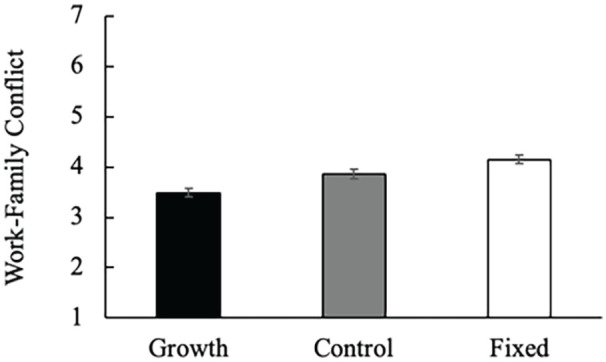
Effect of Condition on Women’s Work–Family Conflict (Study 3). *Note.* Error bars represent standard errors.

We predicted work–family conflict by condition using a one-way analysis of variance (ANOVA). We found a main effect of condition, *F*(2, 700) = 14.33, *p* < .001, η^2^ = .04, 95% CI = [0.02, 0.07], such that women in the growth condition (*M* = 3.49, *SD* = 1.33) reported significantly less work–family conflict than those in the fixed, *M* = 4.15, *SD* = 1.32; *t*(700) = 5.34, *p* < .001, and control, *M* = 3.86, *SD* = 1.37; *t*(700) = 3.00, *p* = .003, conditions. The difference between the fixed and control conditions was also significant, *t*(700) = 2.33 *p* = .02. See Figure 3. These results remained significant without participant exclusions.

#### Behavioral Intentions

**Figure 4.**
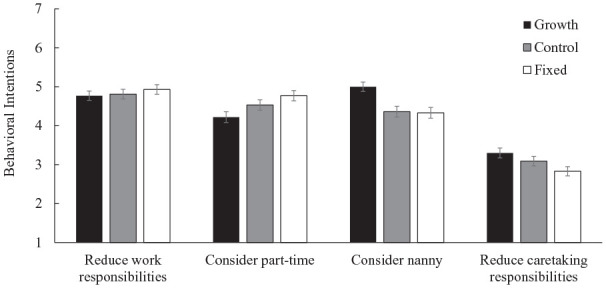
Effect of Condition on Women’s Behavioral Intentions (Study 3). *Note.* Error bars represent standard errors.

We tested the effect of mindset condition on each of the proposed actions using one-way ANOVAs. As depicted in Figure 4, we found a main effect of mindset condition on considering part-time work, *F*(2, 700) = 4.16, *p* = .016, η^2^ = .01, 95% CI = [0.0, 0.03]; hiring a nanny, *F*(2, 700) = 7.92, *p* < .001, η^2^ = .02, 95% CI = [0.01, 0.05]; and cutting back on caretaking responsibilities, *F*(2, 700) = 3.77, *p* = .023, η^2^ = .01, 95% CI = [0.00, 0.03], such that participants in the growth condition reported that they would be less likely to consider going part time at work, *M* = 4.22, *SD* = 2.09; *t*(700) = 2.88, *p* = .004, and more likely to hire a nanny, *M* = 5.00, *SD* = 1.84; *t*(700) = -3.57, *p* < .001, and cut back on caretaking responsibilities, *M* = 3.30, *SD* = 1.92; *t*(700) = -2.74, *p* = .006, compared to the fixed condition (part-time: *M* = 4.77, *SD* = 2.06; nanny: *M* = 4.33, *SD* = 2.14; caretaking: *M* = 2.83, *SD* = 1.84). Participants in the growth and fixed conditions did not significantly vary from those in the control condition in considering part-time work (*M* = 4.53, *SD* = 2.05) or cutting back on caretaking responsibilities (*M* = 3.09, *SD* = 1.88) (*p*s > .11). Participants in the growth condition were more likely to consider hiring a nanny than those in the control condition, *M* = 4.36, *SD* = 2.12; *t*(700) = −3.33, *p* < .001; however, the fixed condition did not significantly differ from the control condition. Participants reported intentions to cut back on work responsibilities did not vary significantly by mindset condition.

### Discussion

In Study 3, we first replicated the effect of gender role mindsets on work–family conflict in an online sample of women. We also demonstrated the directionality of the effect, such that in comparison to our control condition, growth mindsets significantly reduced work–family conflict, while fixed mindsets significantly increased work–family conflict. We then explored possible mechanisms by measuring behavioral intentions in response to a hypothetical scenario involving a high degree of work–family conflict. We found support for our proposed mechanism, prescriptive gender roles. However, because we collected all measures at a single time point, we cannot be certain of the directionality of the pathway. Compared to women in the growth mindset conditions, women in the fixed mindset condition were more likely to consider reducing their hours at work, actions consistent with intensified gender role prescriptions. We also found that, relative to women in the fixed mindset condition, women in the growth condition were more likely to seek help with childcare and caregiving responsibilities, effective strategies for coping with family demands, which corresponded with them anticipating less work–family conflict. Taken together, the results are consistent with our expectations around intensified gender role prescriptions for women holding fixed mindsets and more relaxed gender role prescriptions for women holding growth mindsets.

## Study 4

Building off our results from Studies 1 to 3, the final study explored the relationship between gender role mindsets and work–family conflict for women and men in heterosexual, dual-career couples during the COVID-19 pandemic. While Study 1 measured anticipated work–family conflict, we examined the relationship between gender role mindsets and current work–family conflict in the present study.

Prior research has shown a relationship between conflict and reduced job and family satisfaction (Abdullah et al., 2021; Carlson et al., 2011; Dahm et al., 2015; Eckman, 2004; Ford et al., 2007; Kulik et al., 2016; Matthews et al., 2014), which correlates with workplace withdrawal and voluntary turnover (i.e., Mobley, 1977). Prior researchers have also shown the spillover of work demands on family and relationship satisfaction and likewise family demands on job satisfaction. Thus, we measured both job and relationship satisfaction (Ford et al., 2007; Frone et al., 1992). Our goal was to explore whether growth mindsets can improve satisfaction, given that satisfaction is a precursor to withdrawal and voluntary turnover behavior (Griffeth et al., 2000; Mobley, 1977; Porter et al., 1974). We hypothesized that work–family conflict would mediate the relationship between gender role mindset and job and relationship satisfaction.

### Method

We preregistered work–family conflict to mediate the relationship between gender role mindset and satisfaction measures. Moderation of the mediation by gender was exploratory.^8^

#### Participants

We recruited *n* = 100 heterosexual couples. Requirements for participation were as follows: at least one individual graduated from a large public university undergraduate or graduate program, both individuals were working at least part time, and couples were either married or cohabitating. We ended up with a sample of 98 couples, as within two couples, one individual did not finish the survey. As a result, we had a sample of highly educated and high-earning dual-career couples (95% of participants had at least an undergraduate degree, 52% had a graduate degree, and the average base salary was US$110,375).

#### Measures and Procedure

Participants were emailed a link to the online survey with the following measures (see Supplemental Material for items):

##### Gender Role Mindset

Participants completed the same 10-item gender role mindset measure from Study 1 (α = .89).

##### Work–Family Conflict

Participants completed the same 7-item scale as Studies 2 and 3 (α = .84).

##### Job Satisfaction

Participants completed an 18-item scale and rated whether each item described their employment situation (Balzer et al., 1997). Sample items include “Worthwhile,” “Acceptable,” and “Enjoyable.” The response options were “Yes,” “No,” and “Cannot Decide.” Scores on each item are summed to compute the job satisfaction measure. Higher scores indicated greater experienced job satisfaction (α = .90).

##### Relationship Satisfaction

We measured relationship satisfaction using two previously validated measures: Dyadic Adjustment Scale (DAS) Dyadic Consensus subscale (Spanier, 1976) and Relationship Assessment Scale (RAS) (Hendrick et al., 1998).

Dyadic Adjustment asks participants to rate how much they agree with their partner for each item on a scale from 1 (*always disagree*) to 6 (*always agree*). Sample items include “Handling family finances” and “Career decisions.” Higher scores indicated greater relationship satisfaction (α = .85).

RAS asks participants to assess their relationship with their partner. Sample items include “How well does your partner meet your needs” and “In general, how satisfied are you with your relationship.” The response scale ranged from 1 (*poorly*) to 6 (*extremely well*). Higher scores indicated greater relationship satisfaction (α = .90).

##### Control Variables

We included the identical measures of BE and traditional gender role preference as in Study 1. We also added measures of benevolent and hostile sexism (HS; Glick & Fiske, 2001), which might cause work–family conflict for women who work if they believe they are inferior to men in the workplace and that men should be taking care of them so that they can avoid paid work.

### Results

#### Work–Family Conflict

**Figure 5.**
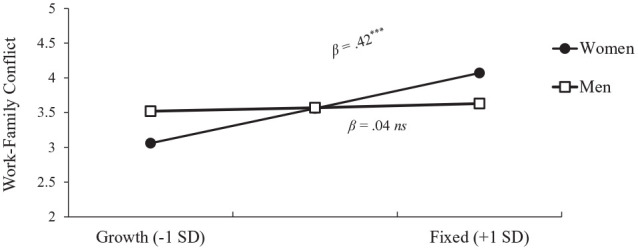
Work–Family Conflict by Gender and Gender Role Mindset in Dual-Career Couples (Study 4). †*p* < .10. *p < .05. **p < .01. ***p < .001.

Table 5 reports descriptive statistics and correlations for all study variables.^9^ We used a multilevel model with random intercepts and included gender, gender role mindset, and Gender Role Mindset × Gender interaction. Individual difference measures were standardized before running the regression. Table 6 summarizes the results of the regressions predicting work–family conflict. We did not find a significant main effect of gender, β = −.01, *t*(191) = −0.07, *p* = .947.^10^ We did find a main effect of gender role mindset, β = .23, *t*(191) = 3.29, *p* = .001, such that more fixed mindsets were correlated with increased work–family conflict. We also found a significant interaction between gender role mindset and gender, β = .38, *t*(190) = 2.82, *p* = .005: Comparing participants with a fixed (+1 *SD*) gender role mindset, women had significantly more work–family conflict than men, β = .37, *t*(133) = 1.98, *p* = .05. Women with growth (−1 *SD*) gender role mindset had significantly less work–family conflict than men with growth gender role mindset, β = −.39, *t*(133) = −2.07, *p* = .04; however, for men, the correlation between mindset and work–family conflict was nonsignificant, β = .04, *t*(194) = 0.45, *p* = .656. It was for women only, that more fixed mindsets were significantly correlated with more work–family conflict, β = .42, *t*(190) = 4.36, *p* < .001. See Figure 5. The effect of gender role mindset on work–family conflict for women remained significant when including control measures in the multilevel model: PTGR, endorsement of BE, HS, and benevolent sexism (BS), and interaction terms with gender.

**Table 5.** Descriptive Statistics and Correlations for Study 4 Variables.

	Men (*N* = 98)		Women (*N* = 98)		Correlations									
Variable	*M*	*SD*	*M*	*SD*	(1)	(2)	(3)	(4)	(5)	(6)	(7)	(8)	(9)	(10)
(1) Gender	—	—	—	—	—									
(2) Gender role mindset	3.0	0.9	2.9	0.9	−.06	—								
(3) Work–family conflict	3.6	1.2	3.5	1.2	−.02	.23**	—							
(4) Job satisfaction	43.6	10.9	39.7	12.7	−.17*	−.14*	−.39***	—						
(5) Relationship Assessment Scale	4.7	0.6	4.7	0.6	.03	−.08	−.26***	.25***	—					
(6) Dyadic Adjustment Scale	59.8	7.3	61.6	6.1	.14†	−.13†	−.28***	.12†	.47***	—				
(7) Biological essentialism	3.2	1.0	2.7	1.1	−.21**	.54***	.28***	−.15*	−.29***	−.17*	—			
(8) Preference for traditional gender roles	1.9	0.7	1.6	0.6	−.26***	.52***	.19**	−.10	−.25***	−.27***	.67***	—		
(9) Benevolent sexism	3.2	0.7	2.8	0.8	−.23**	.36***	.25***	−.05	−.17*	−.25***	.63***	.62***	—	
(10) Hostile sexism	2.5	0.9	2.2	0.8	−.19**	.54***	.27***	−.15*	−.29***	−.28***	.66***	.80***	.60***	—

*Note.* Gender role mindset: Higher values indicate more fixed mindset.

† *p* < .10. *p < .05. **p < .01. ***p < .001.

**Table 6.** Study 4 Regression Analysis of Work–Family Conflict.

Predictors	Model 1					Model 2					Model 3					Model 4				
	β	*SE*	CI	*t*	*p* value	β	*SE*	CI	*t*	*p* value	β	*SE*	CI	*t*	*p* value	β	*SE*	CI	*t*	*p* value
(Intercept)	.00	0.10	[0.19, 0.20]	0.04	.964	.02	0.10	[−0.17, 0.20]	0.17	.866	−.03	0.10	[−0.22, 0.16]	−0.33	.738	−.06	0.10	[−0.25, 0.13]	−0.58	.561
Gender	−.01	0.13	[−0.27, 0.25]	−0.07	.947	−.01	0.13	[−0.26, 0.25]	−0.07	.946	.08	0.13	[−0.17, 0.34]	0.65	.518	.09	0.13	[−0.16, 0.35]	0.73	.467
GRM	.23	0.07	[0.09, 0.37]	3.29	.**001**	.04	0.10	[−0.15, 0.23]	0.45	.656	−.07	0.11	[−0.28, 0.14]	−0.68	.494	−.11	0.12	[−0.34, 0.12]	−0.92	.357
Gender × GRM						.38	0.13	[0.12, 0.64]	2.82	.**005**	.33	0.13	[0.07, 0.59]	2.50	.**012**	.36	0.16	[0.04, 0.68]	2.20	.**027**
PTGR											−.14	0.12	[−0.38, 0.10]	−1.13	.257	−.03	0.16	[−0.34, 0.28]	−0.19	.849
BE											.15	0.10	[−0.05, 0.35]	1.49	.135	.26	0.14	[−0.01, 0.54]	1.87	.062
HS											.17	0.12	[−0.06, 0.40]	1.46	.145	−.02	0.16	[−0.33, 0.29]	−0.11	.914
BS											.12	0.09	[−0.06, 0.30]	1.32	.186	.17	0.13	[−0.09, 0.42]	1.29	.198
Gender × PTGR																−.22	0.24	[−0.69, 0.25]	−0.93	.352
Gender × BE																−.20	0.20	[−0.59, 0.20]	−0.97	.332
Gender × HS																.39	0.23	[−0.06, 0.83]	1.69	.092
Gender × BS																−.07	0.18	[−0.42, 0.28]	−0.38	.707
Marginal *R*^2^/Conditional *R*^2^	.054/.129					.090/.172					.148/.243					.163/.280				
ICC	.08					.09					.11					.14				

*Note.* Gender coded: 0 = man, 1 = woman. Work–family conflict, GRM, PTGR, BE, HS, and BS measures were all standardized. CI = confidence interval; GRM = gender role mindset; PTGR = preference for traditional gender roles; BE = biological essentialism; HS = hostile sexism; BS = benevolent sexism; ICC = intraclass correlation coefficient.

Bolded p-values are statistically significant (p < .05). .

#### Satisfaction

We next tested whether there was an indirect effect of gender role mindset on our satisfaction measures, through work–family conflict. We found that gender role mindsets were not significantly correlated with job satisfaction, DAS, or RAS. However, the indirect effects of gender role mindset on job satisfaction and DAS through work–family conflict were significant (*b*indirect = −.09, 95% CI = [−0.15, −0.03], *z* = −2.78, *p* = .006; *b*indirect = −.06, 95% CI = [−0.12, −0.02], *z* = −2.43 *p* = .015) and marginally significant for RAS (*b*indirect = −.03, 95% CI = [−0.07, −0.004], *z* = −1.84, *p* = .065). Using a mediation model, we found that gender role mindset was positively correlated with work–family conflict, β = .23, *t*(191) = 3.28, *p* = .001. We also found that work–family conflict was negatively correlated with our three satisfaction measures: job satisfaction, DAS, and RAS, β = −.37, *t*(191) = −5.42, *p* < .001; β = −.26, *t*(191) = −3.77, *p* < .001; β = −.14, *t*(191) = −2.36, *p* = .019. Therefore, we found a significant indirect effect of gender role mindset on our three satisfaction measures, through work–family conflict.

#### Moderated Mediation

**Figure 6.**
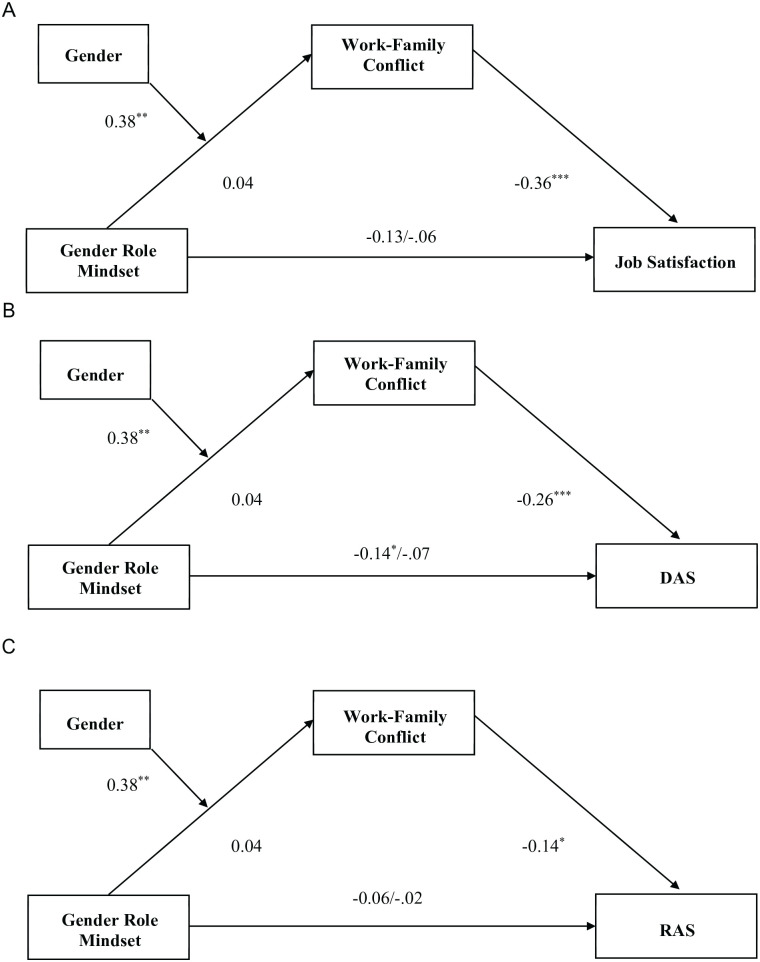
Moderated Mediation on Three Measures of Satisfaction (Study 4). (A) Job Satisfaction. (B) Dyadic Adjustment Scale. (C) Relationship Assessment Scale. *Note.* Gender coded. 0 = men, 1 = women. Higher gender role mindset values indicate a more fixed gender role mindset. DAS = Dyadic Adjustment Scale; RAS = Relationship Assessment Scale. †*p* < .10. *p < .05. **p < .01. ***p < .001.

The moderated mediation model (see Figure 6A–6C) was tested in a single model to assess the significance of the indirect effects at differing levels of the moderator gender. Gender was found to moderate the indirect effect of gender role mindset and work–family conflict, β = .38, *t*(166) = 2.79, *p* = .006. More fixed gender role mindsets predicted higher work–family conflict for women, β = .42, *t*(166) = 4.32, *p* < .001, but were not significantly predictive for men, β = .04, *t*(166) = 0.44, *p* = .660. In turn, higher work–family conflict predicted lower job satisfaction, β = −.36, *t*(190)= −5.47, *p* < .001. Work–family conflict also predicted lower relationship satisfaction, as measured by both scales, DAS, β = −.26, *t*(190)= −3.81, *p* < .001, and RAS, β = −.14, *t*(190)= −2.35, *p* = .02. These moderated mediation results remain significant for job satisfaction and DAS, but not for RAS, when we add PTGR, endorsement of BE, HS, and BS to the model.

### Discussion

To summarize the results of Study 4, we first found that the significant correlation between gender role mindset and anticipated work–family conflict in students observed in Study 1, and in women samples in Studies 2 and 3, extends to current work–family conflict in dual-career couples. Importantly, we also found that for women, gender role mindset is correlated with work–family conflict, which is negatively correlated with satisfaction measures. These results remained significant with the inclusion of related gender constructs, except for our RAS measure. The RAS and DAS are highly correlated; however, the Dyadic Consensus subscale is more specific to relationship conflict and disagreements than the broader RAS measure, which could explain the stronger relationship between DAS and work–family conflict. Understanding the pathway from a more fixed gender role mindset to less satisfaction for women is important, as reduced satisfaction is an antecedent to women leaving the workplace.

## General Discussion

The current research identifies a novel pathway through which mindsets about the fixedness versus malleability of gender roles in society contribute to work–family conflict and reduce job and relationship satisfaction. Across four studies, we found evidence that a fixed gender role mindset blights women’s ability to feel as though they can “have it all.” In particular, a fixed gender role mindset was associated with more work–family conflict for women but not men. In Study 1, a fixed gender role mindset was correlated with college-aged women’s greater anticipation of having to choose between a successful career and a family. Interestingly, these results were found in undergraduate students who have not likely experienced said conflict yet. Given that work–family conflict increases over time as a function of life stages, these results might underestimate the work–family conflict fixed gender role mindset women will later endure (Hall, 1975). In Study 2, using a manipulation of gender role mindsets based on participants’ self-generated examples of ways in which gender roles are fixed versus malleable, we demonstrated the causal impact of holding the belief that gender roles can change on reducing women’s work–family conflict. In Study 3, we demonstrated that growth gender role mindsets lead to more relaxed gender role prescriptions and reduced work–family conflict for women. In our final study, which was conducted during COVID-19, a period that exposed the embeddedness of gender roles and created additional challenges for dual-career couples, we found a consistent pattern linking fixed gender role mindset to current work–family conflict in high-earning dual-career couples. Further, we demonstrated the negative correlation between women’s heightened work–family conflict and job and relationship satisfaction, which prior research has linked to employee turnover and withdrawal (i.e., Carsten & Spector, 1987).^11^

### Theoretical and Practical Implications

Our research makes theoretical contributions to several areas of the literature. First, we showed that gender role mindsets uniquely affect women’s work–family conflict, by controlling for several theoretically relevant constructs across our set of studies. We demonstrated that work–family conflict is not explained by a simple PTGR, which might contribute to working women’s conflict for those who prefer traditional roles of domestic over paid labor. Next, we controlled for alternative essentialism theories that purport biological differences between men and women determine their behaviors and outcomes. In addition to an established measure of BE, we also measured gender determinism, a construct that theoretically touches on implicit personality theory, but whose items resemble that of essentialist theories (e.g., “gender basically determines an individual’s behaviors”). Women working outside the home who hold more essentialist beliefs could have greater work–family conflict, as doing so violates biological imperatives for child-rearing. While each of these constructs is related, we believe work–family conflict, which results from competing role demands, is best understood through the lens of gender role mindset, which measures the extent that people hold the belief that these roles can change. While we know of few interventions to shift other gender beliefs such as essentialism, idiosyncratic preferences, and sexism, research shows some success with interventions designed to foster more of a growth mindset (Dweck, 2006; Yeager et al., 2019). That said, we did not expect our effect to be explained by broader implicit personality beliefs either, given that work–family conflict results precisely from traditional gender role expectations. We demonstrated that interventions, aimed at instilling a growth *gender role mindset* specifically, reduce women’s work–family conflict and may promote a greater sense of satisfaction among women, at work and in their relationships.

We also revisited and integrated prior literature on conflict, satisfaction, and turnover, demonstrating gender role mindset is a novel antecedent to both a holistic sense of work–family conflict (i.e., whether one can “have it all”), as well as directional conflict stemming from work interfering with family and family interfering with work (Ford et al., 2007; Frone et al., 1992). In addition, while mindsets have been a fruitful area of research for decades, gender role mindsets are a relatively new construct that is still being understood. Kray et al. (2017) showed unique effects of gender role mindset on men’s (but not women’s) system justification; the current research demonstrated novel effects of gender role mindset on women’s (but not men’s) work–family conflict. For men, we consistently found that gender role mindset is uncorrelated with work–family conflict. These patterns suggest gender role mindsets are relevant to both men and women, and that there are unique downstream consequences for each gender.

### Limitations and Future Research

Like all research, a limitation of our data is the context in which it was collected. First, we focused on traditional gender roles, assuming heterosexual relationships. Because of this, we can only speculate as to how these results would generalize to other sexual orientations. Some research suggests same-sex couples exhibit more equality in sharing domestic tasks, which is associated with less marital strain (Garcia & Umberson, 2019). Same-sex couples might serve as role models of growth mindset behaviors to the benefit of heterosexual couples seeking more egalitarian arrangements and is an important direction for future research.

Another contextual factor is that all our participants were in the United States. Historical gender roles in the United States may differ from other countries, and therefore so do what the malleability of gender roles implies. Specifically, self-stereotyping along traditional gender roles is greater in Western cultures (Guimond et al., 2007), which may carry implications for how strongly gender role mindsets influence women’s work–family conflict. Women in countries in which gender roles are more equitable than the United States may not feel as constrained by a fixed gender role mindset, as their existing gender role system is not as prescriptive as in the United States. Future researchers should partake in cross-cultural comparisons of the relationship between gender role mindset and work–family conflict. In addition, in the United States, there are also structural barriers to changing gender roles, and the onus should not be placed entirely on women. While we do find that inducing a growth gender role mindset reduces women’s anticipated work–family conflict, lasting change in gendered divisions of labor and work–family conflict require systemic changes.

We also acknowledge that our research does not provide for a fine-grained analysis of the intersectional effects of gender by race or social class. In addition, certain behaviors, such as the ability to hire a nanny (Study 3), are not financially feasible options for all women. Motherhood expectations, and the resulting penalties, are based on White middle-class families, in which White mothers are perceived negatively for working outside the home (Rosette et al., 2018). However, Black women are expected to work and are perceived as less hardworking when they are stay-at-home mothers (Cuddy & Wolf, 2013). Black and Hispanic women incur less of a wage penalty than White women when they become mothers (Budig & England, 2001; Glauber, 2007). Separately, prior research has shown that Asian women may also escape a motherhood penalty, perhaps mitigated by the model minority stereotype (Denny, 2014). Thus, due to the intersection of gender and racial stereotypes, the pressure of gendered expectations and resulting work–family conflict likely varies across racial groups. Understanding work–family conflict through an intersectional lens is an important future direction of this research.

### Conclusion

Implicit theories about how fixed or malleable gender roles are can have serious implications for gender equity. In this research, we demonstrated the impact that these mindsets have on anticipated and experienced work–family conflict. Progress is slowly being made, and gender role attitudes have become more egalitarian over time (Donnelly et al., 2016). Nevertheless, we posit that a fixed gender role mindset hinders women’s progress via work–family conflict, as identified in the present research. Thus, progress may lie in espousing a growth gender role mindset.

## Supplemental Material

sj-docx-1-psp-10.1177_01461672231178349 – Supplemental material for Holding the Belief That Gender Roles Can Change Reduces Women’s Work–Family Conflict Supplemental material, sj-docx-1-psp-10.1177_01461672231178349 for Holding the Belief That Gender Roles Can Change Reduces Women’s Work–Family Conflict by Charlotte Townsend, Laura Kray and Alexandra Russell in Personality and Social Psychology Bulletin

## Notes

1. See Supplemental Material for sample size justifications and exclusions.

2. Across studies, statistics were done using R 4.1.1 (R Core Team, 2020; RStudio Team, 2019), the reghelper (v2.0.2; Hughes & Beiner, 2021), dplyr (v1.0.7; Wickham et al., 2021), ggplot2 (v3.3.5; Wickham, 2016), Hmisc (v4.6-0; Harrell, 2021), pwr (v1.3-0; Champely, 2020), effectsize (v0.6.0.1; Ben-Shachar et al., 2020), sjPlot (v2.8.9; Lüdecke et al., 2021), lme4 (v1.1.27.1; Bates et al., 2021), simr (v1.0.6; Green & MacLeod, 2016), bruceR (v0.7.2; Bao, 2021), and MuMIn (v1.46.0; Bartoń, 2022) packages.

3. We previously attempted a scientific article manipulation—see Supplemental Material.

4. This is a replication of an experiment provided in the Supplemental Material.

5. The results are also significant controlling for political ideology across studies. See Supplemental Material.

6. We also included behavioral intention measures focused on collaborating with others at work and at home to find solutions to work–family conflict. See Supplemental Material.

7. We preregistered a *t* test between experimental conditions.

8. We preregistered several additional analyses and measures which are not included. See Supplemental Material for analysis of similarity of gender role mindsets.

9. Based on our sample size, we were underpowered to detect partner and similarity effects. These analyses are included in Supplemental Material.

10. One post hoc explanation for the lack of significant difference is that most of the couples do not yet have children.

11. We also find men’s mindsets, and the similarity between mindsets within the couple, are uncorrelated with women’s reported work–family conflict. However, we were also underpowered to detect partner and similarity effects.
