# Supplementary material for: Holding the Belief That Gender Roles Can Change Reduces Women’s Work–Family Conflict
Source: Pers Soc Psychol Bull. 2023 Jun 18;50(11):1613–32. doi: 10.1177/01461672231178349 (PMC11504165; doi:10.1177/01461672231178349)
Supplement: sj-docx-1-psp-10.1177_01461672231178349 – Supplemental material for Holding the Belief That Gender Roles Can Change Reduces Women’s Work–Family Conflict [file sj-docx-1-psp-10.1177_01461672231178349.docx]

**Supplemental Materials**

**Holding the Belief that Gender Roles Can Change Reduces Women’s Work-Family Conflict**

Table of Contents

| Study 1 Measures……………………………………………………………....... | 2 |
| --- | --- |
| Participant Sample and Exclusions for Studies 1-3...…………………………… | 4 |
| Studies 2 - 3 Manipulation Text………...……………………………………..... | 5 |
| Alternative Article Manipulation………………………………………………... | 7 |
| Table A1.………………………………………………………………... | 10 |
| Figure A1………………………………………………………………... | 11 |
| Alternative Article Manipulation Text….………….…………………… | 12 |
| Supplemental Experiment for Study 2…………………………………………... | 14 |
| Table A2.………………………………………………………………... | 16 |
| Figure A2………………………………………………………………... | 17 |
| Additional Statistical Analysis for Political Ideology Across Studies…………... | 18 |
| Study 3 Additional Statistical Analysis…………………………………………. | 19 |
| Study 4 Additional Measures……………………………………………………. | 21 |
| Study 4 Additional Statistical Analysis….………………….…………………... | 24 |
| Table A3.……………………………………………………………….... | 25 |
| Table A4.……………………………………………………………….... | 25 |
| References……………………………………………………………………….. | 26 |

**Study 1 Measures**

Study 1 was part of a larger survey distributed to undergraduate students for course credit. Demographic questions including gender were included in the larger survey, the composites below were those used for this specific study.

**Endorsement of Biological Essentialism (Brescoll et al., 2013, adapted from Keller, 2005)**

Please rate the extent to which you disagree or agree with the following statements. (1 Strongly Disagree – 6 Strongly Agree)

1. I think that differences between men and women in personality are largely determined by genetic factors
2. I think that differences between men and women in behavior are largely determined by the biological differences between the genders
3. Part of the reason why women are more emotional than men is because of the way they're hard-wired
4. I believe that men pursue math and science careers more than women because of the innate difference between the genders
5. Men commit the majority of violent crimes in this country because they have a greater predisposition towards violence than women
6. Women get in fewer physical fights than men because men have less of an inborn tendency for aggression
7. I think that the reason why there are more male math professors than female math professors is due to some biological differences between the sexes

**Preference for Traditional Gender Roles (Larsen & Long’s (1998) 20-item Attitudes Toward Sex Roles Scale)**

Please rate the extent to which you disagree or agree with the following statements. (1 Strongly Disagree – 6 Strongly Agree)

1. A man who has chosen to stay at home and be a house-husband is not less masculine.
2. It is just as important to educate daughters as it is to educate sons.
3. Women should be more concerned with clothing and appearance than men.
4. Women should have as much sexual freedom as men.
5. The man should be more responsible for the economic support of the family than the woman.
6. The belief that women cannot make as good supervisors or executives as men is a myth.
7. The word "obey" should be removed from wedding vows.
8. Ultimately a woman should submit to a husband's decision.
9. Some equality in marriage is good, but by and large the husband ought to have the main say-so in family matters.
10. Having a job is just as important for a wife as it is for her husband.
11. In groups that have both male and female members, it is more appropriate that leadership positions be held by males.
12. I would not allow my son to play with dolls.
13. Having a challenging job or career is just as important as being a wife and mother.
14. Men make better leaders.
15. Almost any woman is better off in her home than in a job or profession.
16. A woman's place is in the home.
17. The role of teaching in the elementary schools belongs to women.
18. The changing of diapers is the responsibility of both parents.
19. Men who cry have weak character.
20. As head of the household, the father should have the final authority over the children.

**Participant Sample and Exclusions for Studies 1-3**

**Study 1.** The sample size was determined by course enrollment so we conducted sensitivity analysis in g*power and found that for this sample the required effect size *f* is .25 (greater than our observed effect).

**Study 2.** Based on the effect size of the experiment provided in the Supplemental Materials with the same manipulation, we would need a sample of at least 220 for 0.8 power. We increased the sample size to 500 because we were measuring multiple dependent variables. Based on preregistered criteria^^[[1]](#footnote-1)^^, we excluded 11 participants whose responses were less than 100 characters, and 6 participants who indicated they were either not women or heterosexual. Exclusions did not significantly differ by condition (*p* = .21).

**Study 3.** We increased the sample size from Study 2 because we added a Control condition. Therefore, we still had approximately 250 participants per condition. Based on preregistered criteria^^[[2]](#footnote-2)^^, we excluded 27 participants whose responses were less than 100 characters, and 16 participants who indicated they were either not women or heterosexual. Exclusions did not significantly differ between growth and fixed conditions (*p* = .25)^^[[3]](#footnote-3)^^.

**Studies 2 - 3 Manipulation Text**

**What Are Gender Roles?**

The term "gender roles" refers to divisions of household labor, job segregation, and gender differences in status and authority.

Gender roles influence how people are expected to act, speak, dress, and conduct themselves based on their gender.

Within heterosexual couples, traditional gender roles dictate:

- Women should take care of the home; men should provide for their family.
- Women are supposed to be polite and nurturing; men are expected to be bold and strong.

Text from growth gender role mindset condition:

**Gender Roles Have Changed Across Time**

You have been randomly assigned to consider the ways that **gender roles have changed in your lifetime**. Your first task is to **reflect on a time in which you observed or experienced** **how gender roles have changed.**

Here are examples from previous study participants:

"*Yes!!! My daughter (my oldest) watched me struggle as a single mom and 3 children, working as a secretary at a community college. My daughter didn't finish college (she was three classes short!), but she blazed her way up the chain and is now working at the main office for one of the major banks in the US as a VP. She just didn't let her gender stop her.*"

"*I do feel that roles are changing and that it is very acceptable that women have a career and men are home raising children. I think gender roles have changed in that there has been an increase in male nurses, caretakers and child raisers.*"

"*I thought it was impossible to have children and work. But my daughter made sure she had someone to come in and clean once a week and had a live-in nanny. (I just have trouble imagining doing that!) So she didn't work 50 hours a week and then come home to clean the house and be a housewife. She and her husband are both professionals making over $200k each. They both cleaned and cooked and took care of the children. They just were smart about what they did. I wish it was something I could have done!*"

Text from fixed gender role mindset condition:

**Gender Roles Are Stable Across Time**

You have been randomly assigned to consider the ways that **gender roles have stayed the same in your lifetime**. Your first task is to **reflect on a time in which you observed or experienced how gender roles have persisted.**

Here are examples from previous study participants:

"*I would love to say that women don't have to sacrifice a career in order to pursue a family. Unfortunately, especially with the pandemic, this has not been my experience. My husband is also extremely liberal and is a feminist. Due to job and financial stability however he is the one that has been able to further his career path and I am the one that is currently at home with our children.*"

"*Women suffer at the hands of men who use a woman to do household duties and deprive her of having a meaningful life outside of serving her husband and kids. Even in today's age with women working, they are still expected to carry the burden of all the housework while the man just sits back because he is "so tired from a 40 hour workweek".*"

"*I saw firsthand that you cannot have both a full-time job & be an effective hands-on parent. So, I made the decision to be a full-time parent & my kids turned out better for it than my brother & myself. If you try to do both, you're only doing both halfway & you're letting someone else raise your kids or else the kids are raising themselves. When I hear someone say that they can be a great parent & they work full-time, I don't say anything, but I know they're fooling themselves & down the road, they'll see the negative impact of not choosing one or the other.*"

**Alternative Article Manipulation**

We attempted to manipulate mindsets using a scientific article, a common method for influencing mindsets (Levy et al., 1998; Rattan & Ozgumus, 2019). Due to the unique correlation between gender role mindset and work-family conflict in women in Study 1, we only recruited women participants.

**Method**

The methods, sample size, and analysis plan for this study were preregistered (<https://aspredicted.org/KWJ_BBT>).

**Participants**. We recruited *n* = 1000 online college-educated women participants in the United States from Prolific who self-identified as heterosexual to reflect our participants from Study 1. We ended up with 1009 participants when the study completed. Based on preregistered criteria, we excluded 4 participants who failed both of the attention checks, and 56 participants who indicated they were either not women or heterosexual or college educated. All participants in our study completed the survey during the COVID-19 pandemic in May 2021. A prior G*Power analysis confirmed we were well-powered to detect the effect based on the effect size in Kray et al. (2017)(Faul et al., 2009). The majority of our participants were employed, full-time (57.8%), part-time (11.9%), or self-employed (9.0%), and the remaining participants were either students (4.5%), unemployed (5.2%), homemakers (6.3%), or retired (5.4%). Our participants also ranged in years of work experience: less than 1 year (9.3%), 2-5 years (19.8%), 5-10 years (23.4%), 10-20 years (23.8%), and 20+ years (23.7%). Ethnic composition was as follows: White (73.3%), Asian (11.6%), Black (6.7%), Hispanic/Latinx (4.3%), Multiracial (3.1%), Other (0.6%), and East Asian (0.3%). Average age was *M* = 38.08 years old*, SD* = 12.97.

**Measures and procedure**. Participants were given a link to the online survey in which they were assigned to read one of two articles, intended to instill either or growth or a fixed mindset. We used a gender role mindset article manipulation taken from Kray et al. (2017), based on a paradigm developed to manipulate theories of intelligence (Chiu, Hong, & Dweck, 1997). For example, in the growth mindset article:

In a recent paper (Smith & Wilson, 2020) summarizing a wide range of longitudinal studies that address this question it was determined that the vast majority of society’s division of labor between the sexes is due to **environmental factors that can change over time**.

The same portion in the fixed mindset article reads:

In a recent paper (Smith & Wilson, 2020) summarizing a wide range of longitudinal studies that address this question it was determined that the vast majority of society’s division of labor between the sexes is due to **personal factors that remain stable over time**.

After reading the article, participants were given two attention check questions and asked to summarize the article, followed by a section to evaluate the article on a variety of measures. Next, participants completed the work-family conflict composite and then demographic questions.

***Article evaluations.*** Participants completed five questions about the article after the task. Participants rated each article from -3 (*Very Ineffective*) to 7 (*Very Effective*) on Accuracy, Writing clarity, Persuasiveness, Introduction of new knowledge, and Neutrality (α = .84).

***Work-family conflict.*** Participants completed the same 7-item scale as Study 2, (α = .87).

**Results**

Contrary to our hypothesis, we did not find a significant difference in work-family conflict across mindset conditions (*β* = -.03, *t*(947) = -.40, *p* = .69). However, in an exploratory fashion, we tested for moderation by article evaluations. We find significant moderation of the effect of mindset condition on women’s work-family conflict by article evaluations (*β* = .19, *t*(945) = 2.46, *p* = .01, *f* = 0.08): in the fixed condition, higher article evaluations were significantly positively correlated with work-family conflict (*β* = .13, *t*(945) = 2.73, *p* = .006). However, in the growth condition, article evaluations were not significantly correlated with work-family conflict, but directionally negatively associated with work-family conflict (*β* = -.06, *t*(945) = -1.03, *p* = .30). See Figure A1. The moderation remains significant (*β* = .19, *t*(937) = 2.38, *p* = .02) when run simultaneously with other possible moderators (age, political ideology, highest level of education attained, years of work experience). See Table A1.

**Discussion.** We used a “fake news” scientific article manipulation consistent with previous mindset manipulations, however, we did not find a main effect of mindset condition on work-family conflict. It is possible in the last few years with the rise of ‘fake news’ and criticism of scientific research, the effectiveness of the scientific article manipulation has weakened. It is also possible that the essay manipulation is only effective on men, given Kray et al. (2017) observed changes in gender system justification for men but not women on the basis of this identical manipulation. In the present study that only includes women, we found that the more positively the article was viewed, exposure to the fixed gender role mindset essay predicts more work-family conflict. Similarly, provided the growth essay was positively evaluated, the less work-family conflict was reported. Yeager and Dweck (2020) recently argued that more work is needed to understand situational moderators of mindset interventions, and this study addresses this call by providing direction for mindset researchers who may wish to use scientific articles as a manipulation in the future, with the caveat that they may need to preregister this potential moderator.

Table A1

*Regression Analysis of Work-Family Conflict by Condition and Moderators for Alternative Article Manipulations*

|  | **Model 1** | | | | | **Model 2** | | | | | **Model 3** | | | | | | **Model 4** | | | | | |
| --- | --- | --- | --- | --- | --- | --- | --- | --- | --- | --- | --- | --- | --- | --- | --- | --- | --- | --- | --- | --- | --- | --- |
| *Predictors* | *β* | *SE* | *CI* | *t* | *p* | *β* | *SE* | *CI* | *t* | *p* | *β* | *SE* | *CI* | *t* | *p* | *β* | | *SE* | *CI* | *t* | *p* |  |
| (Intercept) | 3.75 | 0.06 | [3.63, 3.87] | 62.70 | **<0.001** | 3.45 | 0.20 | [3.05, 3.85] | 16.96 | **<0.001** | 4.08 | 0.33 | [3.44, 4.73] | 12.46 | **<0.001** | 4.82 | | 0.76 | [3.33 – 6.31 | 6.33 | **<0.001** |  |
| Mindset Condition | -0.03 | 0.08 | [-0.20, 0.13] | -0.40 | 0.687 | 0.00 | 0.09 | [-0.17, 0.18] | 0.06 | 0.955 | -0.94 | 0.39 | [-1.72, -0.17] | -2.39 | **0.017** | -1.41 | | 1.06 | [-3.49, 0.67] | -1.33 | 0.185 |  |
| Article Evaluation |  |  |  |  |  | 0.06 | 0.04 | [-0.02, 0.13] | 1.56 | 0.119 | -0.06 | 0.06 | [-0.18, 0.06] | -1.03 | 0.304 | -0.07 | | 0.06 | [-0.20, 0.05] | -1.17 | 0.242 |  |
| Mindset Condition x Article Evaluation |  |  |  |  |  |  |  |  |  |  | 0.19 | 0.08 | [0.04, 0.34] | 2.46 | **0.014** | 0.19 | | 0.08 | [0.03, 0.34] | 2.38 | **0.018** |  |
| Age |  |  |  |  |  |  |  |  |  |  |  |  |  |  |  | -0.02 | | 0.01 | [-0.03, -0.00] | -2.17 | **0.031** |  |
| Political id |  |  |  |  |  |  |  |  |  |  |  |  |  |  |  | -0.01 | | 0.02 | [-0.05, 0.04] | -0.27 | 0.790 |  |
| Education |  |  |  |  |  |  |  |  |  |  |  |  |  |  |  | -0.06 | | 0.12 | [-0.30, 0.18] | -0.48 | 0.631 |  |
| Employment experience |  |  |  |  |  |  |  |  |  |  |  |  |  |  |  | 0.08 | | 0.07 | [-0.06, 0.22] | 1.09 | 0.276 |  |
| Mindset Condition x Age |  |  |  |  |  |  |  |  |  |  |  |  |  |  |  | 0.03 | | 0.01 | [0.01, 0.05] | 2.49 | **0.013** |  |
| Mindset Condition x Political Id |  |  |  |  |  |  |  |  |  |  |  |  |  |  |  | -0.04 | | 0.03 | [-0.11, 0.03] | -1.15 | 0.251 |  |
| Mindset Condition x Education |  |  |  |  |  |  |  |  |  |  |  |  |  |  |  | 0.11 | | 0.18 | [-0.24, 0.45] | 0.61 | 0.544 |  |
| Mindset Condition x Experience |  |  |  |  |  |  |  |  |  |  |  |  |  |  |  | -0.24 | | 0.10 | [-0.44, -0.04] | -2.34 | **0.020** |  |
| Observations | 949 | | | | | 949 | | | | | 949 | | | | | | 949 | | | | | |
| R^2^ / R^2^ adjusted | 0.000 / -0.001 | | | | | 0.003 / 0.001 | | | | | 0.009 / 0.006 | | | | | | 0.023 / 0.012 | | | | | |

**Fig. A1*.*** Work-Family Conflict by Article Evaluation and Mindset Condition.

**Alternative Article Manipulation Text**

Article from Growth Mindset manipulation:
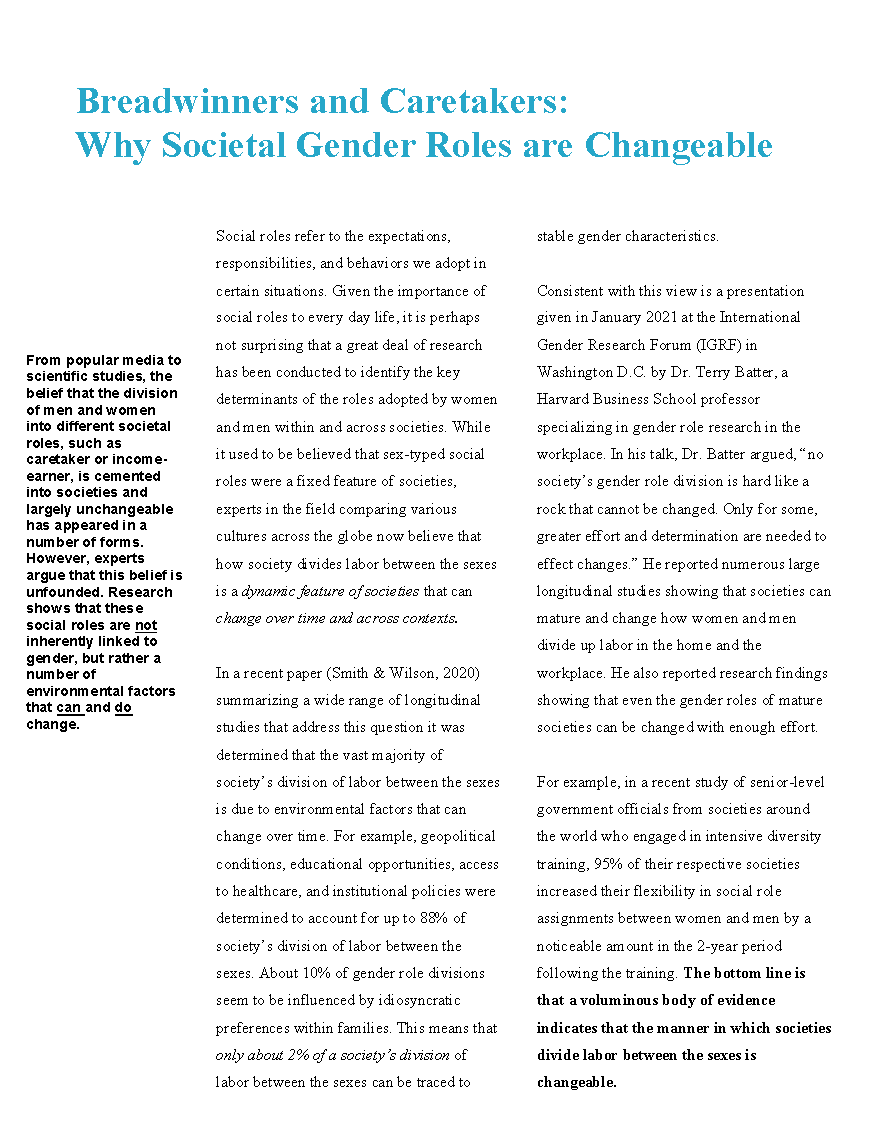


Article from Fixed Mindset manipulation:


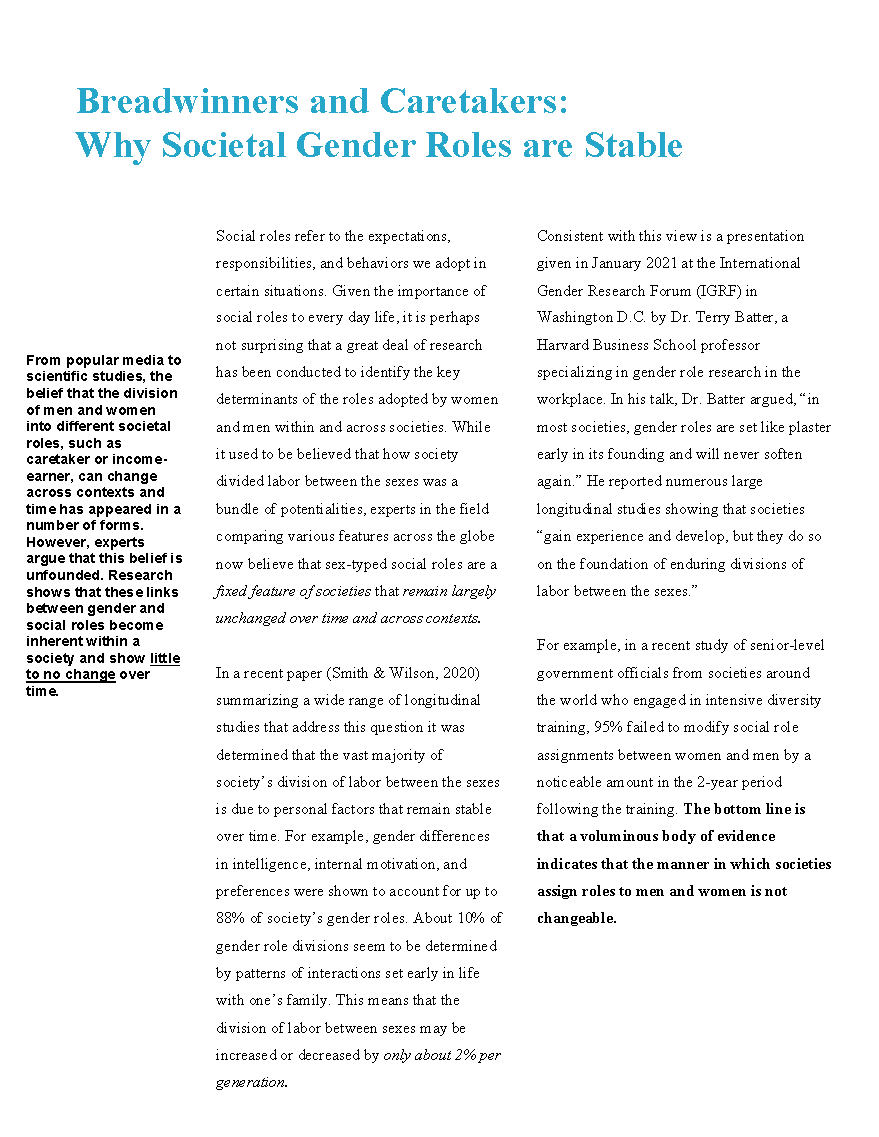


**Supplemental Experiment for Study 2**

We used an experimental design to demonstrate the causal relationship between gender role mindsets and work-family conflict.

**Method**

The methods, sample size, and analysis plan for this study were preregistered (https://aspredicted.org/L9K_KKT). We pre-registered that women in the fixed condition would report more work-family conflict than women in the growth condition.

**Participants**. We recruited *n* = 500 online women participants in the United States from Prolific who self-identified as heterosexual and ended up with 501 participants when the study completed. Based on preregistered criteria, we excluded 21 participants whose responses were less than 100 characters, 2 participants who wrote nonsensical answers, and 13 participants who indicated they were either not women or heterosexual.^^[[4]](#footnote-4)^^ Exclusions did not significantly differ by condition (*p* = .97). See Table A2 for participant demographic information.

**Measures and procedure**. Participants were given a link to the online survey in which they were assigned to reflect and write about either how gender roles have changed or how they have persisted, intended to instill either a growth or a fixed mindset from the outset of the survey. We based this gender role mindset reflection manipulation on work that leverages mindset self-reflection (Heslin et al., 2005; Wilson, 1990). For example, the growth (fixed) condition read:

You have been randomly assigned to consider the ways that **gender roles have changed (stayed the same) in your lifetime**. Your first task is to **reflect on a time in which you observed or experienced** **how gender roles have changed (persisted).**

After describing their personal experience, participants were told to reflect on why gender roles have changed or persisted and what the implications are, followed by our manipulation check and a distractor measure. Next, participants completed the work-family conflict composite and then demographic questions.

***Manipulation check.*** Participants indicated, “To what degree have gender roles changed versus remained the same over time?” from 1 (*Gender roles have remained the same*) to 7 (*Gender roles have changed a lot*). This item was then reverse-coded, so that higher values indicate more fixed beliefs.

***Work-family conflict.*** Participants completed the same 7-item scale as Study 1 but worded in present tense (α = .88).

**Results**

***Manipulation check.*** We found a significant difference between conditions, such that participants in the fixed condition (*M* = 3.7, *SD* = 1.3) more strongly believed that gender roles have remained unchanged than growth condition participants (*M* = 2.2, *SD* = 1.0, *t*(463) = 13.64, *p* < .001, *d* = 1.27, 95% CI [1.07, 1.47]).

***Work-family conflict.*** We predicted work-family conflict by condition using a linear regression. We found a main effect of condition, such that women in the growth condition (*M* = 3.5, *SD* = 1.3) reported significantly less work-family conflict than in the fixed condition (*M* = 4.0, *SD* = 1.3) (*β* = .38, *t*(463) = 4.15, *p* < .001, *d* = .38, 95% CI [.20, .57]). See Figure A2. These

results remain significant without participant exclusions.^[[5]](#footnote-5)^

**Discussion.** In this study, we demonstrated the causal relationship between gender role mindsets and work-family conflict for women. Specifically, we found that women who are situationally induced to hold a fixed gender role mindset report greater work-family conflict than women who are situationally induced to hold a growth gender role mindset.

|  | **(N=465)** |
| --- | --- |
| **Employment Status** |  |
| Employed full-time | 202 (43.4%) |
| Employed part-time | 62 (13.3%) |
| Unemployed | 28 (6.0%) |
| Self-employed | 45 (9.7%) |
| Homemaker | 68 (14.6%) |
| Student | 32 (6.9%) |
| Retired | 28 (6.0%) |
| **Ethnicity** |  |
| White/Caucasian | 348 (74.8%) |
| Asian/Asian-American | 31 (6.7%) |
| Black/African American | 55 (11.8%) |
| Hispanic/Latino | 14 (3.0%) |
| East Asian/East Asian American | 1 (0.2%) |
| Multiracial | 11 (2.4%) |
| Other | 5 (1.1%) |
| **Age** |  |
| Mean (SD) | 38.3 (13.4) |
| Median [Min, Max] | 34.0 [18.0, 79.0] |

Table A2. *Demographics for participants.*

**Fig. A2.** Effect of Condition on Women’s Work-Family Conflict


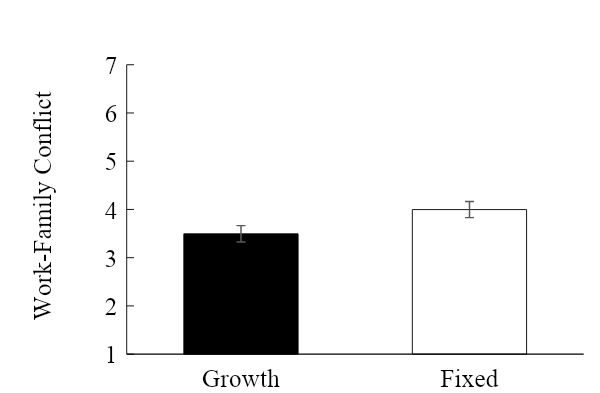


Error bars represent standard errors.

**Additional Statistical Analysis for Political Ideology Across Studies**

In Study 1, we did not collect the participant’s political ideology. In Study 2, the effect of mindset condition is significant when controlling for political ideology, and political ideology x mindset condition is not significant (Study 2: *β* = .005, *t*(479)=.04, *p* = .97). In Study 3, the effect of mindset condition is also significant when controlling for political ideology, and the gender x political ideology interaction is not significant (*F*(2, 697) = .15, *p* = .86). In Study 4, when we control for political ideology and add the interaction term gender x political ideology, the gender x gender role mindset interaction remains significant, political ideology is not significantly correlated with work-family conflict, and the gender x political ideology interaction is marginally significant (*β* = .28, *t*(152)=1.85, *p* = .07).

**Study 3 Additional Statistical Analyses**

We included the following additional composites of work-focused and family-focused behaviors as exploratory mechanisms:

***Work-focused behavioral intentions.*** Participants indicated how likely they would be to do each of the following work-focused behaviors: “Talk with coworkers about my conflicts between work and nonwork.”, “Take the time to share information with my coworkers about my personal needs.”, “Explore how my job can be organized to jointly benefit employees and the company.”, “Offer suggestions to make it easier for employees to balance work and nonwork demands.”, “Propose reallocating job duties creatively to help my department work better as a team.”, and “Collaborate with others at work as a whole team to enable everyone's needs to be met.” The response scale ranged from 1 (*Extremely unlikely*) to 7 (*Extremely likely*). Higher scores indicated a greater likelihood of taking said action. We analyzed the items as a composite (α = .83).

***Family-focused behavioral intentions.*** Participants indicated how likely they would be to do each of the following family-focused behaviors: “Talk with family members about my conflicts between work and nonwork.”, “Take the time to share information with my family about my work-related needs.”, “Explore how my family can be organized to jointly benefit everyone.”, “Offer suggestions to my family to make it easier to balance work and nonwork demands.”, “Propose reallocating job duties creatively to help my family work better as a team.”, and “Collaborate with my family as a whole team to enable everyone's needs to be met.” The response scale ranged from 1 (*Extremely unlikely*) to 7 (*Extremely likely*). Higher scores indicated a greater likelihood of taking said action. We analyzed the items as a composite (α = .82).

**Results**

***Work-focused behavioral intentions.*** We test the effect of mindset condition on the work-focused coping strategy composite using a one-way ANOVA. We do not find a main effect of condition (*F*(2,700) = .43, *p* = .65).

***Family-focused behavioral intentions.*** We test the effect of mindset condition on the family-focused coping strategy composite using a one-way ANOVA. We do not find a main effect of condition (*F*(2,700) = 2.29, *p* = .10).

**Discussion**

We do not find a significant difference in work-focused or family-focused behavioral intentions by gender role mindset condition. One noteworthy difference between these behaviors and the behavioral intentions we report in the main text, is that these behaviors are interpersonal whereas the behaviors in the main text are personal. It is possible that our mindset manipulation affects women’s decisions as they pertain to themselves, but not when they require agreement from others. This coincides with what we find in Study 4, that only women’s gender role mindset is correlated with work-family conflict, and not their partner’s nor the similarity between their gender role mindsets. We also note that these work-focused and family-focused behaviors include discussing work-family conflict and personal needs, whereas the items in the main text do not refer explicitly to discussions around work-family conflict and personal needs.

**Study 4 Additional Measures**

**Hostile Sexism (Glick & Fiske, 2001)**

Please rate the extent to which you disagree or agree with the following statements. (1 Strongly Disagree – 6 Strongly Agree)

1. Many women are actually seeking special favors, such as hiring policies that favor them over men, under the guise of asking for “equality”.
2. Most women interpret innocent remarks or acts as being sexist.
3. Women are too easily offended.
4. Feminists are not seeking for women to have more power than men.
5. Most women fail to appreciate fully all that men do for them.
6. Women seek to gain power by getting control over men.
7. Women exaggerate problems they have at work.
8. Once a woman gets a man to commit to her, she usually tries to put him on a tight leash.
9. When women lose to men in a fair competition, they typically complain about being discriminated against.
10. There are actually very few women who get a kick out of teasing men by seeming sexually available and then refusing male advances.
11. Feminists are making entirely reasonable demands of men.

**Benevolent Sexism (Glick & Fiske, 2001)**

Please rate the extent to which you disagree or agree with the following statements. (1 Strongly Disagree – 6 Strongly Agree)

1. No matter how accomplished he is, a man is not truly complete as a person unless he has the love of a woman.
2. In a disaster, women ought not necessarily to be rescued before men.
3. People are often truly happy in life without being romantically involved with a member of the other sex.
4. Many women have a quality of purity that few men possess.
5. Women should be cherished and protected by men.
6. Every man ought to have a woman whom he adores.
7. Men are complete without women.
8. A good woman should be set on a pedestal by her man.
9. Women, compared to men, tend to have a superior moral sensibility.
10. Men should be willing to sacrifice their own well-being in order to provide financially for the women in their lives.
11. Women, as compared to men, tend to have a more refined sense of culture and good taste.

**Job Satisfaction (Baltzer et al., 1997)**

Does this item describe your employment situation over the past year? (Yes, No, Cannot Decide)

- Pleasant
- Bad
- Ideal
- Waste of time
- Good
- Undesirable
- Worthwhile
- Worse than most
- Acceptable
- Superior
- Better than most
- Disagreeable
- Makes me content
- Inadequate
- Excellent
- Rotten
- Enjoyable
- Poor

**Dyadic Adjustment Scale – Dyadic Consensus (Spanier, 1976)^^[[6]](#footnote-6)^^**

Most people have disagreements in their relationships. Please indicate below the approximate extent of agreement or disagreement between you and your partner for each item on the following list, over the past year. (1 Always Disagree – 6 Always Agree)

1. Handling family finances
2. Matters of recreation
3. Religious matters
4. Friends
5. Conventionality (correct or proper behavior)
6. Philosophy of life
7. Ways of dealing with parents or in-laws
8. Aims, goals, and things believed important
9. Amount of time spent together
10. Making major decisions
11. Household tasks
12. Leisure time interests and activities
13. Career decisions

**Relationship Assessment Scale (Hendrick et al., 1998)**

Please answer the following questions about your relationships with your partner over the past year as accurately as possible. (1 – 5)

1. How well does your partner meet your needs?
2. In general, how satisfied are you with your relationship?
3. How good is your relationship with your partner compared to most?
4. How often do you wish you hadn’t gotten into this relationship?
5. To what extent does your relationship meet your original expectations?
6. How much do you love your partner?
7. How many problems are there in your relationship?

**Study 4 Additional Statistical Analysis**

We first used Response Surface Analysis (RSA) to examine the link between gender role mindset and work-family conflict.^^[[7]](#footnote-7)^^ To examine the role of actor gender role mindset, partner gender role mindset, and gender role mindset similarity^^[[8]](#footnote-8)^^, we added the polynomial regression parameters to the APIM:

*Work-family conflict = b_0_ + b_1_X_actor_ + b_2_Y_partner_+b_3_X_actor_^2^+b_4_X_actor_Y_partner_+b_5_Y_partner_^2^*
In this equation, work-family conflict was predicted by actor gender role mindset X_actor_, partner gender role mindset Y_partner_, their interaction term X_actor_Y_partner_, and their quadratic terms X_actor_^2^ and Y_partner_^2^ (Weidmann et al., 2017). The three second-order terms (X_actor_^2^, X_actor_Y_partner_, and Y_partner_^2^) together can reflect similarity effects, which can be tested when using RSA.  We do not find a significant effect of similarity of gender role mindset nor a partner effect, so we simplified the model to a multilevel model in the main text.^^[[9]](#footnote-9)^,^[[10]](#footnote-10)^^ See Tables A3 and A4 for results.

Table A3. Cross-sectional dyadic polynomial regression coefficients of both partners’ Gender Role Mindsets on **women's** work-family conflict and satisfaction.

|  | **Work-Family Conflict** | **Work-Family Conflict with Controls** | **Job Satisfaction** | **Dyadic Adjustment** | **Relationship Assessment** |
| --- | --- | --- | --- | --- | --- |
| b_1_ actor rating | 0.46^***^ [0.18, 0.75] | 0.34^*^ [0.01, 0.66] | 0.07  [-0.19, 0.33] | -0.04  [-0.32, 0.24] | 0.16  [-.14, 0.47] |
| b_2_ partner rating | 0.05 [-0.19, 0.29] | 0.01 [-0.25, 0.27] | 0.06  [-0.15, 0.26] | 0.09  [-0.11, 0.29] | 0.07  [-0.15, 0.29] |
| b_3_ actor rating^2^ | -0.24 [-0.53, 0.06] | -0.26 [-0.57, 0.05] | 0.31^**^  [0.12, 0.51] | 0.22^y^  [0.00, 0.44] | 0.05  [-0.17, 0.26] |
| b_4_ actor rating x partner rating | 0.11 [-0.24, 0.46] | 0.13 [-0.24, 0.50] | -0.24^y^  [-0.48, 0.00] | -0.05  [-0.34, 0.24] | -0.10  [-0.39, 0.19] |
| b_5_ partner rating^2^ | 0.01 [-0.24, 0.26] | -0.004 [-0.24, 0.23] | 0.12  [-0.08, 0.32] | 0.04  [-0.20, 0.28] | 0.04  [-0.21, 0.28] |
| cv_1_ Preference for Traditional Gender Roles |  | -0.002 [-0.49, 0.49] | 0.00  [-0.37, 0.38] | -0.39 ^y^  [-0.81, 0.02] | -0.33^y^  [-0.67, 0.01] |
| cv_2_ Biological Essentialism |  | 0.2 [-0.15, 0.55] | -0.13  [-0.43, 0.16] | -0.03  [-0.33, 0.26] | -0.23  [-0.50, 0.05] |

Notes. Polynomial regression coefficients (b_1_  b_5_) are unstandardized b-weights but due to the pooled-standardization across partners, they can be interpreted as standardized b-weights. 95% confidence intervals are given in brackets []. ^y^ p < 0.10. *p < 0.05. **p < 0.01. ***p < 0.001.

Table A4. Cross-sectional dyadic polynomial regression coefficients of both partners’ Gender Role Mindsets on **men’s** work-family conflict and satisfaction.

|  | **Work-Family Conflict** | **Work-Family Conflict with Controls** | **Job Satisfaction** | **Dyadic Adjustment** | **Relationship Assessment** |
| --- | --- | --- | --- | --- | --- |
| b_1_ actor rating | 0.03 [-0.26, 0.32] | -0.20 [-0.59, 0.19] | -0.20  [-0.46, 0.05] | 0.16  [-0.14, 0.45] | 0.27^*^  [0.06, 0.48] |
| b_2_ partner rating | 0.12 [-0.12, 0.36] | 0.09 [-0.15, 0.34] | -.08  [-0.34, 0.18] | -0.11  [-0.33, 0.12] | -.00  [-0.19, 0.18] |
| b_3_ actor rating^2^ | -0.03 [-0.36, 0.31] | 0.01 [-0.32, 0.31] | -0.02  [-0.20, 0.16] | 0.36^**^  [-0.48, 0.19] | 0.24^y^  [-0.01, 0.49] |
| b_4_ actor rating x partner rating | 0.05 [-0.31, 0.41] | 0.08 [-0.29, 0.44] | -0.01  [-0.30, 0.27] | -0.14  [-0.48, 0.19] | -0.20  [-0.47, 0.08] |
| b_5_ partner rating^2^ | 0.13 [-0.12, 0.38] | 0.15 [-0.13, 0.42] | -0.17  [-0.45, 0.12] | -0.01  [-0.21, 0.20] | 0.19^*^  [0.00, 0.37] |
| cv_1_ Preference for Traditional Gender Roles |  | 0.03 [-0.44, 0.49] | -0.06  [-0.43, 0.31] | -0.10  [-0.51, 0.30] | -0.07  [-0.43, 0.30] |
| cv_2_ Biological Essentialism |  | 0.36^*^ [0.04, 0.68] | -0.10  [-0.37, 0.16] | -0.01  [-0.25, 0.23] | -0.35  [-0.59, -0.10] |

Notes. Polynomial regression coefficients (b_1_  b_5_) are unstandardized b-weights but due to the pooled-standardization across partners, they can be interpreted as standardized b-weights. 95% confidence intervals are given in brackets []. ^y^ p < 0.10. *p < 0.05. **p < 0.01. ***p < 0.001.

**References**

Baltzer, W. K., Kihm, J. A., Smith, P. C., Irwin, J. L., Bachiochi, P. D., & Robie, C. (1997). *Users’ manual for the Job Descriptive Index (JDI; 1997 Revision) and the Job In General scales.*

Brescoll, V. L., Uhlmann, E. L., & Newman, G. E. (2013). The effects of system-justifying motives on endorsement of essentialist explanations for gender differences. *Journal of Personality and Social Psychology*, *105*(6), 891–908. https://doi.org/10.1037/a0034701

Chiu, C., Hong, Y., & Dweck, C. S. (1997). Lay dispositionism and implicit theories of personality. Journal of Personality and Social Psychology, 73(1), 19–30. https://doi.org/10.1037/0022-3514.73.1.19

Faul, F., Erdfelder, E., Buchner, A., & Lang, A.-G. (2009). Statistical power analyses using G*Power 3.1: Tests for correlation and regression analyses. Behavior Research Methods, 41(4), 1149–1160. https://doi.org/10.3758/BRM.41.4.1149

Glick, P., & Fiske, S. T. (2001). An ambivalent alliance: Hostile and benevolent sexism as complementary justifications for gender inequality. *American Psychologist*, *56*, 109–118.

Heslin, P. A., Latham, G. P., & VandeWalle, D. (2005). The effect of implicit person theory on performance appraisals. *Journal of Applied Psychology*, *90*(5), 842–856. https://doi.org/10.1037/0021-9010.90.5.842

Humberg, S., Nestler, S., & Back, M. D. (2019). Response Surface Analysis in Personality and Social Psychology: Checklist and Clarifications for the Case of Congruence Hypotheses. *Social Psychological and Personality Science*, *10*(3), 409–419. https://doi.org/10.1177/1948550618757600

Keller, J. (2005). In genes we trust: The biological component of psychological essentialism and its relationship to mechanisms of motivated social cognition. *Journal of Personality and Social Psychology*, *88*(4), 686–702. https://doi.org/10.1037/0022-3514.88.4.686

Kray, L. J., Howland, L., Russell, A., & Jackman, L. M. (2017). The effects of implicit gender role theories on gender system justification: Fixed beliefs strengthen masculinity to preserve the status quo. *Journal of Personality and Social Psychology*, *112*, 98–115.

Larsen, K. S., & Long, E. (1988). Attitudes toward sex-roles: Traditional or egalitarian? *Sex Roles*, *19*(1), 1–12. <https://doi.org/10.1007/BF00292459>

Levy, S. R., Stroessner, S. J., & Dweck, C. S. (1998). Stereotype formation and endorsement: The role of implicit theories. *Journal of Personality and Social Psychology*, *74*(6), 1421–1436. <https://doi.org/10.1037/0022-3514.74.6.1421>

Rattan, A., & Ozgumus, E. (2019). Embedding mindsets in context: Theoretical considerations and opportunities for studying fixed-growth lay theories in the workplace. *Research in Organizational Behavior*, *39*, 100127. https://doi.org/10.1016/j.riob.2020.100127

Spanier, G. B. (1976). Measuring dyadic adjustment: New scales for assessing the quality of marriage and similar dyads. *Journal of Marriage and Family*, *38*(1), 15–28. https://doi.org/10.2307/350547

Weidmann, R., Schönbrodt, F. D., Ledermann, T., & Grob, A. (2017). Concurrent and longitudinal dyadic polynomial regression analyses of Big Five traits and relationship satisfaction: Does similarity matter? *Journal of Research in Personality*, *70*, 6–15. <https://doi.org/10.1016/j.jrp.2017.04.003>

Wilson, T. D. (1990). Self-persuasion via self-reflection. In J. M. Olson, M. P. Zanna, & C. P. Herman, *Self-Inference Processes: The Ontario Symposium, Volume 6*. Psychology Press.

Yeager, D.S., & Dweck, C. S. (2020). What can be learned from growth mindset controversies? *The American Psychologist, 75*(9), 1269-1286. <https://doi.org/10.1037/amp0000794>

1. We also pre-registered to exclude participants who wrote nonsensical answers which did not result in any exclusions. [↑](#footnote-ref-1)
2. We also pre-registered to exclude participants who wrote nonsensical answers which did not result in any exclusions. [↑](#footnote-ref-2)
3. Exclusions varied significantly across conditions, as more participants were excluded in the Control condition due to writing less than 100 characters. [↑](#footnote-ref-3)
4. We conducted sensitivity analysis in g*power and found that for this sample the required effect size *f* is .13 (smaller than our observed effect). [↑](#footnote-ref-4)
5. The effect of mindset condition is significant when controlling for political ideology, and political ideology x mindset condition is not significant (*β* = .002, *t*(461)=.03, *p* = .97). [↑](#footnote-ref-5)
6. We pre-registered items 1-15 of the Dyadic Adjustment Scale but the analyses included are the Dyadic Consensus subscale. The two items: Demonstrations of affection and Sex relations are part of the Affectional Expression subscale. The results remain significant if we adhere to the pre-registration and include these two items. [↑](#footnote-ref-6)
7. We preregistered a variable-centered approach and joint mindsets but conducted RSA as this is a better test of similarity (Humberg et al., 2019). [↑](#footnote-ref-7)
8. We preregistered similarity analyses. [↑](#footnote-ref-8)
9. We followed procedures similar to previous research on personality and only interpreted polynomial and response surface regression coefficients that were significant at a *p* < .01 level (Weidmann et al., 2017). [↑](#footnote-ref-9)
10. Based on our sample size, we were underpowered to detect partner and similarity effects. [↑](#footnote-ref-10)
